# Potential Involvement of Platelet-Derived Microparticles and Microparticles Forming Immune Complexes during Monocyte Activation in Patients with Systemic Lupus Erythematosus

**DOI:** 10.3389/fimmu.2018.00322

**Published:** 2018-03-01

**Authors:** Catalina Burbano, Juan Villar-Vesga, Janine Orejuela, Carlos Muñoz, Adriana Vanegas, Gloria Vásquez, Mauricio Rojas, Diana Castaño

**Affiliations:** ^1^Grupo de Inmunología Celular e Inmunogenética, Instituto de Investigaciones Médicas, Facultad de Medicina, Universidad de Antioquia UdeA, Medellín, Colombia; ^2^Unidad de Citometría de Flujo, Sede de Investigación Universitaria, Universidad de Antioquia UdeA, Medellín, Colombia; ^3^Sección de Reumatología, Hospital Universitario San Vicente Fundación, Medellín, Colombia

**Keywords:** microparticles, monocyte activation, immune complexes, systemic lupus erythematosus, monocyte subsets

## Abstract

Microparticles (MPs) are vesicles derived from the plasma membrane of different cells, are considered a source of circulating autoantigens, and can form immune complexes (MPs-ICs). The number of MPs and MPs-ICs increases in patients with systemic lupus erythematosus (SLE). MPs activate myeloid cells by inducing IL-6 and TNF-α in both SLE and other diseases. Therefore, we propose that the recognition of MPs-ICs by monocytes rather that MPs may define their phenotype and contribute to the inflammatory process in patients with SLE. Thus, the aims of this study were to evaluate the association among circulating MPs-ICs from different cell sources, alterations observed in monocyte subsets, and disease activity in patients with SLE and to establish whether monocytes bind and respond to MPs-ICs *in vitro*. Circulating MPs and monocyte subsets were characterized in 60 patients with SLE and 60 healthy controls (HCs) using multiparametric flow cytometry. Patients had higher MP counts and frequencies of MPs-CD41a + (platelet-derived) compared with HCs, regardless of disease activity. MPs from patients with SLE were C1q + and formed ICs with IgM and IgG. MPs-IgG + were positively correlated with active SLE (aSLE), whereas MPs-IgM + were negatively correlated. Most of the circulating total ICs-IgG + were located on MPs. The proportion and number of non-classical monocytes were significantly decreased in patients with SLE compared with HCs and in patients with aSLE compared with patients with the inactive disease. Non-classical monocytes obtained from patients with SLE exhibited increased levels of CD64 associated with MPs-IgG +, MPs-C1q +, total circulating ICs-IgG +, and disease activity. The direct effects of MPs and MPs-IgG + on monocytes were evaluated in cell culture. Monocytes from both HCs and patients bound to and internalized MPs and MPs-IgG + independent of CD64. These vesicles derived from platelets (PMPs), mainly PMPs-IgG +, activated monocytes *in vitro* and increased the expression of CD69, CD64, and pro-inflammatory cytokines such as IL-1β, TNF-α, and IFN-α. Therefore, MPs are one of the most representative sources of the total amount of circulating ICs-IgG + in patients with SLE. MPs-IgG + are associated with SLE activity, and PMPs-IgG + stimulate monocytes, changing their phenotype and promoting pro-inflammatory responses related to disease activity.

## Introduction

Circulating autoantibodies against different nuclear components are one of the main serological hallmarks of systemic lupus erythematosus (SLE). These autoantibodies can form immune complexes (ICs) in the circulation, which are then deposited at the intravascular level, inducing inflammatory processes and tissue damage (1). Different sources of autoantigens have been implicated in the formation of ICs in patients with SLE, including apoptotic bodies (2) that contain autoantigens such as DNA and modified histones (3), and, more recently, microparticles (MPs) (4). MPs are vesicles derived from plasma membranes of activated and apoptotic cells (ACs). Therefore, similar to ACs, MPs are associated with different autoantigens, such as autoantigens derived from nucleosomes (5) and have a broad spectrum of effects on immune responses (6). For example, monocytes and macrophages seem to play critical roles in the clearance of these structures (7–9), and monocyte-derived MPs induced reactive oxygen species (ROS) production, the release of cytokines, such as IL-6, and NF-κB activation in human monocytes and macrophages (10). Additionally, MPs from apoptotic neutrophils from healthy donors together with raised IFN-α concentrations induced the secretion of the pro-inflammatory cytokines IL-6, IL-8, and TNF-α in monocytes from the same donors, and also macrophage polarization was shifted toward a more inflammatory type after the same treatment (11). In other phagocytic cells, it has been observed that endothelial-derived MPs induced plasmacytoid dendritic cell maturation, in addition to upregulation of costimulatory molecules and inflammatory cytokines secretion (IL-6 and IL-8) (12).

Increased numbers of circulating MPs have been reported in patients with SLE (13). MPs can compete with ACs for removal by binding to scavenger receptors (SRs) (14). In addition, these structures or their contents may be subject to posttranslational modifications, including citrullination and oxidation, which contribute to alarmin and neoantigen availability (15). The MPs containing acetylated chromatin drive ROS-independent neutrophil extracellular trap (NET) release in cells from patients with SLE-presenting active nephritis (16). All these factors, together with the presence of intracellular components on the MP surface, such as nucleic acids (17), enable these vesicles to bind to the toll-like receptors (TLRs) present on phagocytes (18–20). This recognition contrasts with the non-inflammatory responses generated by SRs and may promote the classical activation of these cells. MPs derived from ACs from patients with SLE increase the expression of the costimulatory molecules CD40, CD80, and CD86 and the production of pro-inflammatory cytokines IL-6, TNF-α, and IFN-α in dendritic cells (21).

As shown in the study by Nielsen et al., circulating MPs are associated with IgG and IgM antibodies (MPs-ICs) as well as complement molecules in patients with SLE (4). Soluble ICs have been directly related to activation of phagocytes, the inflammatory process, and tissue damage in patients with SLE and murine models of lupus (22–25). MPs-IgG + can provide additional signals to myeloid cells through Fc gamma receptors (FcγRs), promoting their pro-inflammatory responses (26). However, MPs-IgM + and MPs-C1q + are also recognized by receptors that induce alternative activation of these cells (27, 28). Therefore, we propose that circulating MPs-ICs rather than MPs may modulate and even define the functional profile of monocytes in patients with SLE and contribute to some extent to the perpetuation and exacerbation of the inflammatory process in patients with SLE, in a similar way than soluble ICs.

Currently, researchers have not clearly identified the mechanisms by which MPs and MPs-ICs affect monocyte subsets in patients with SLE (29); however, alterations in the number and function of these cells as well as changes in the contents and number of MPs have been described independently in these patients (4, 30, 31). In addition, the activation of monocytes by MPs has been observed in different diseases and models including SLE; however, this aspect needs to be solved for MPs-ICs. Therefore, our aims were to establish correlations among circulating MPs-ICs from different cell sources, alterations observed in monocyte subsets, and disease activity in patients with SLE, and to establish whether MPs-CIs bind to and activate monocytes *in vitro*.

## Materials and Methods

### Reagents, Materials, and Antibodies

RPMI-1640 medium supplemented with GlutaMAX, Dulbecco’s phosphate-buffered saline (DPBS), and fetal bovine serum (FBS) were purchased from Gibco-BRL (Grand Island, NY, USA). Paraformaldehyde, RNase A (DNase- and protease-free), and DNase I (RNase-free supplied with MnCl2) were acquired from Thermo Fisher Scientific, Inc. (Pittsburgh, PA, USA). Histopaque^®^-1077, Collagen Type IV, trypan blue, dimethyl sulfoxide anhydrous ≥99.9% (DMSO), ionomycin from *Streptomyces conglobatus*, and lipopolysaccharide (LPS) from *Escherichia coli* O111:B4 were obtained from Sigma-Aldrich (St. Louis, MO, USA). Penicillin and streptomycin were purchased from Cambrex-BioWhittaker (Walkersville, MD, USA). Propidium iodide (PI), Annexin-V, Annexin-V binding buffer, lysis solution, the BD^TM^ Human Inflammatory Cytometric Bead Array (CBA), and BD FACSFlow^TM^ were purchased from BD Pharmingen (San Diego, CA, USA). The sterile polystyrene 12 mm × 75 mm tubes were acquired from BD Falcon (San Diego, CA, USA). Absolute counting beads were obtained from Beckman Coulter (Hialeah, FL, USA). The Fluoresbrite Calibration Grade Size Range Kit (YG calibration grade spheres with diameters of 0.5, 1.0, 2.0, 3.0, and 6.0 µm), Fluoresbrite YG Microspheres (0.1 µm), and Fluoresbrite YG Microspheres (0.2 µm) were acquired from Polysciences, Inc. (Warrington, PA, USA). The Rosette Sep Human Monocyte Enrichment Cocktail was obtained from STEMCELL Technologies (British Columbia, Vancouver, Canada).

Monoclonal antihuman MY4 (CD14)-FITC (Clone 322 A-1) and MY4-RD1 (Clone 322 A-1) antibodies were obtained from Beckman Coulter; the antibodies against human CD16-V450 (Clone 3G8), CD32-PE (Clone 3D3), CD64-APC (Clone 10.1), CD33-P3 (Clone WM-53), CD36-APC (Clone CB38, also known as NL07), CD68-PE (Clone Y1/82A), CD11a-PE-Cy7 (Clone HI111), CD11b-PE (Clone D12), CD11c-PE (Clone B-ly6), CD18-APC (Clone 6.7), FcμR-Alexa Fluor 647 (Clone HM14-1), CD54-PE-Cy5 (Clone HA58), CD41a-PE (Clone HIP8), CD3-PE (Clone 17 A1), CD33-PB (Clone WM53), CD105-BV421 (Clone 266), CD19-V450 (Clone HIB19), CD63-FITC (Clone H5C6), and CD69-PE (Clone FN50) were acquired from BD Biosciences (San Diego, CA, USA). HLA-DR-APC-Cy7 (Clone L243), CD35-PE (E11), and CD93-PE (VIMD2) antibodies were obtained from Biolegend (San Diego, CA, USA). Monoclonal antihuman C1q-FITC (Clone ab4223) and anti-citrulline primary antibodies (Clone ab100932) and the anti-rabbit IgG H&L-Alexa Fluor 647 secondary antibody (Clone ab150079) were obtained from Abcam (San Francisco, CA, USA). The *F*(*ab*’)_2_ anti-IgG fragment conjugated to Alexa Fluor 488 and *F*(*ab*’)_2_ anti-IgM fragment conjugated to Alexa Fluor 647 were purchased from Jackson ImmunoResearch (Baltimore Pike, West Grove, PA, USA). Antihuman CD235a-APC (Clone HIR2) was obtained from eBioscience (San Diego, CA, USA).

### Patients and Controls

Sixty patients with SLE, who were diagnosed according to the American College of Rheumatology criteria (32), were recruited at the Rheumatology Service of “Hospital Universitario San Vicente Fundación” (HUSVF), Medellin, Colombia. Patients were classified according to the SLEDAI (systemic lupus erythematosus disease activity index selena modification) scores as either having inactive SLE (iSLE) (SLEDAI < 4) or active SLE (aSLE) (SLEDAI ≥ 4) (33); the damage index for SLE was measured using the Systemic Lupus International Collaborating Clinics (SLICC) index (33). The main demographic and clinical characteristics of the study subjects were recorded from their medical histories and are shown in Table 1. As controls, we included 60 healthy controls (HCs) with the same gender and similarly ages; additionally, a cohort control of 40 patients with rheumatoid arthritis (RA) were included to compare only some characteristics of MPs and monocyte subsets; these patients were diagnosed according to the American College of Rheumatology and European League Against Rheumatism criteria (34). Our study complies with the Declaration of Helsinki; the research protocol and informed consent form were approved by the Universidad de Antioquia’s Medical Research Institute and HUSVF Ethics Committees. All patients and HCs provided consent and signed the informed consent form.

**Table 1:** Demographic and clinical characteristics of the patients with systemic lupus erythematosus (SLE) and healthy controls (HCs) included in the present study.

	HCs	SLE	
		*iSLE (SLEDAI* < *4)*	*aSLE (SLEDAI* ≥ *4)*
*n*	60	28	32
Age^a^	35 (19–76)	28 (18–73)	28 (18–53)
Sex F/M	53/7	24/4	30/2
Leukocyte count––cells/μL^a^	6,545 (3,250–11,550)	5,968 (1,093–13,255)	5,720 (1,271–12,948)
SLEDAI score^a^	–	0 (0–2)	7 (4–33)
SLICC score^a^	–	0 (0–2)	0 (0–7)
Disease duration––ages^a^	–	7 (0–25)	3 (1–18)
Disease manifestations^b^	–		
Vasculitis		0	5 (16)
Alopecia		0	7 (22)
Malar rash		4 (14)	4 (13)
Butterfly rash		0	1 (3)
Mucosal ulcers		0	3 (9)
Leukopenia		4 (14)	6 (19)
Lymphopenia		5 (18)	16 (50)
Thrombocytopenia		0	7 (22)
Cardiac manifestations		1 (4)	10 (31)
Pulmonary manifestations		0	6 (19)
Neuropsychiatric manifestations		4 (14)	8 (25)
Renal manifestations		15 (54)	27 (84)
Antiphospholipid syndrome		1 (4)	3 (9)
Raynaud’s phenomenon		0	1 (3)
Arthritis		0	6 (19)
Myopathy		1 (4)	3 (9)
ANAS^b^	–	22 (79)	31 (97)
Anti-dsDNA		8 (25)	14 (44)
Other autoantibodies^c^		6 (19)	15 (47)
Low complement^b,d^	–	8 (29)	23 (72)
Therapies^b^	–		
Prednisolone <7.5 mg daily		13 (46)	0
Prednisolone ≥7.5 to ≤30 mg daily		6 (21)	17 (53)
Prednisolone >30 mg daily		2 (7)	12 (38)
Methylprednisolone		7 (25)	15 (47)
Chloroquine, hydroxychloroquine		23 (82)	21 (66)
Azathioprine		5 (18)	4 (13)
Cyclophosphamide		10 (36)	12 (38)
Cyclosporine		0	0
Methotrexate		2 (7)	3 (9)
Mycophenolate mofetil		6 (21)	8 (25)

*^a^Median (minimum–maximum range)*.

*^b^*n* (frequency)*.

*^c^Anti-histone, anti-ENA, anti-C1q, anti-Ro, anti-La, and anti-SM*.

*^d^C3 and C4*.

### Isolation, Counting, Sizing, and Characterization of Microparticles (MPs)

Microparticles were isolated from platelet-poor plasma (PPP) obtained from 4 mL of venous blood treated with the anticoagulant citrate in BD-Vacutainer tubes, as previously described (13). Briefly, immediately after collection, blood cells were removed by centrifugation at 1,800 *× g* for 10 min at 21°C, followed by two centrifugation steps at 3,000 *× g* for 20 min at 21°C to deplete platelets and to obtain PPP. Finally, 1 mL of PPP was centrifuged at 16,900 *×* *g* for 60 min at 21°C to obtain MPs. The MP pellet was suspended in 1 mL of filtered FACS sheath fluid. Polystyrene spheres of known sizes (0.1, 0.5, 1, 2, 3, and 6 µm) were used as a reference to determine the MP size by flow cytometry based on FSC-A and SSC-A parameters, and ultra-filtrated sheath fluid (0.1-µm asymmetric polyethersulfone membrane filters, Thermo Fisher Scientific) was used to set the threshold in the flow cytometer. Some experiments were performed in the presence of the detergent 0.05% Triton X-100 to ensure the vesicular nature of the acquired events. Reference counts of fluorespheres of known concentrations were used to determine the MP concentration in 1 mL of PPP; all events corresponding to MPs in 200 µL of PPP were acquired at a constant low flow and counted on a flow cytometer. The number of events obtained was corrected by the dilution factor. MPs were acquired on a FACS Canto^TM^ II flow cytometer (Becton Dickinson, San Jose, CA, USA) with FACS DIVA software. The percentage and number of MPs were analyzed and estimated using FlowJo 7.6.1 software (Tree Star, Inc., Ashland, OR, USA).

### Cell Source and Phenotype of MPs

Microparticles (200 µL of the MP suspension) were labeled for the flow-cytometry analysis with different panels of specific antibodies (at previously titrated volumes) at room temperature in the dark for 20 min. The MP cell source was determined with monoclonal antibodies against human CD14, CD16, CD41a, CD45, CD105, CD33, CD235a, CD3, CD19, and HLA-DR. An *F*(*ab*’)_2_ anti-IgG fragment conjugated with Alexa Fluor 488 and an *F*(*ab*’)_2_ anti-IgM fragment conjugated with Alexa Fluor 647 were used to determine whether MPs form ICs. A specific primary antibody, an APC-labeled secondary antibody, and a FITC-labeled specific antibody were used to evaluate the presence of citrullinated peptides and C1q on MPs. After staining, a wash with 800 µL of DPBS followed by centrifugation at 16,900*× g* for 60 min at 4°C was performed to remove the excess antibody. MPs were stained with Annexin-V in the presence of Annexin-binding buffer for 20 min at room temperature to assess phosphatidylserine (PS) exposure. The percentages of RNA + and DNA + MPs were determined by exposing those structures to 20 UI of DNase and 1 µg of RNase for 1 h at 37°C; the MPs were subsequently stained with PI (% MPs-DNA + = % MPs PI + −% MPs PI + treated with RNase, and % MPs-RNA + = % MPs PI + −% MPs PI + treated with DNase). This technique only allows the detection of nucleic acids present on the surface of MPs. The FACS Canto^TM^ II flow cytometer with the FACS DIVA software was used to acquire 50,000 MPs; the fluorescence minus one (FMO) method was performed for each fluorochrome to determine the positive and negative events. Freeze-dried powders of antibodies [anti-CD14 and *F*(*ab*)_2_ fractions against IgG and IgM] were centrifuged (14,000*× g*/10 min) after their hydration and before use to avoid noise and non-specific signals from protein aggregates.

### Determination of Total Circulating Immune Complex (IC) and Anti-C1q Antibody Levels

Serum levels of anti-C1q antibodies were measured using an enzyme-linked immunosorbent assay, according to the manufacturer’s instructions (ELISA, Inova, San Diego, CA, USA).

A homemade ELISA was used to determine the concentration of total circulating ICs-IgG +. The ELISA for the total circulating ICs was performed using a previously described method (35), with minor modifications. Briefly, flat bottom polystyrene microtiter plates (MaxiSorp, Thermo Fisher Scientific, Inc.) were coated with 0.5-µg/mL C1q (Sigma-Aldrich) in DPBS overnight at room temperature. Plates were washed three times with DPBS containing 0.05% Tween 20 (DPBST, pH 7.2–7.4) and blocked with 2% BSA (IgG-free and protease-free, Jackson ImmunoResearch) in DPBST for 1–2 h. The serum and plasma samples were diluted 1:1,000–1:10,000 in reagent diluent (HRP Sample Diluent. Inova Diagnostics, Inc., San Diego, CA, USA) and incubated for 2 h. The Peroxidase AffiniPure Goat Anti-Human IgG, Fcγ fragment-specific detection antibody (Jackson ImmunoResearch) was diluted 1:5,000 and incubated with the samples for 60 min at room temperature. After a 20-min incubation with the substrate solution (R&D Systems, Minneapolis, MN, USA), the developed color reaction was stopped with 2N H_2_SO_4_. The absorbance of the samples was immediately recorded at 450 nm in an ELISA plate reader (EL_X_800_NB_, BIO-TEK Instruments, Inc., Winooski, VT, USA). The calibration curve was optimized with heat-aggregated IgG (*r*^2^ ≥ 0.98), which had been previously isolated from the plasma of healthy individuals by affinity chromatography. Serial dilutions of this purified human IgG were aggregated in DPBS at 63°C for 30 min. Negative (samples from healthy individuals and IgG without any artificial aggregation) and positive (samples from patients with aSLE) controls were included in all measurements.

In some cases, the diluted plasma was centrifuged at 16,900*× g* for 60 min at 21°C to determine the extent to which MPs contributed to the total amount of circulating ICs-IgG +. The pellet containing the MPs and the supernatant containing the soluble ICs were evaluated separately using the protocol described above.

### Leukocyte Counts and Monocyte Subset Classification

Total peripheral blood treated with the anticoagulant EDTA was stained with antihuman CD45, CD14, CD16, and HLA-DR for 20 min at room temperature in the dark; FMO controls were performed for each antibody. Red cells were lysed with 1X lysis solution according to the manufacturer’s instructions, and 100,000 cells were acquired immediately on a FACS Canto^TM^ II flow cytometer with FACS DIVA software. The monocyte subsets were defined by FACS based on the surface expression of CD14, CD16, and HLA-DR, as previously reported (29, 36). Leukocyte (CD45 +) counting was performed by the flow cytometer using counting beads and manually with a hemocytometer, as previously described (31). Monocyte subsets were categorized into three subsets based on previous reports (37): CD14 + + CD16− (classical), CD14 + CD16 + + (non-classical), and CD14 + + CD16 + (intermediate) monocytes. The percentage of monocyte subsets were estimated as described for MPs.

### Isolation of Peripheral Blood Mononuclear Cells for the Immunophenotyping of Monocyte Subsets

Peripheral blood mononuclear cells (PBMCs) were obtained from venous blood treated with the anticoagulant EDTA after centrifugation on Histopaque-1077 at 900*× g* for 30 min at room temperature. PBMCs were washed with DPBS and washing buffer (DPBS plus 1% BSA and 0.01% NaN_3_) and suspended in blocking buffer (DPBS plus 1% BSA, 0.01% NaN_3_, and 10% inactivated fetal calf serum). Viability was determined by the exclusion of trypan blue (≥98%). Antibodies against the following molecules were used to determine the expression of molecules associated with MP recognition and mononuclear phagocyte function on monocyte subsets (basic panel CD14, CD16, and HLA-DR): CD64, CD32, CD35, or CD93, CD36, CD11a, CD11b, CD11c, CD18, and CD54. FMO controls were performed for each antibody using the basic panel. Cells were incubated 30 min at 4°C, followed by two additional washes with washing buffer. Fifty thousand monocytes were immediately acquired on a FACS Canto^TM^ II flow cytometer with FACS DIVA software. The percentage of stained cells and the MFI were estimated using FlowJo 7.6.1 software.

### Generation of Platelet-Derived MPs (PMPs)

Platelets were isolated from the whole blood of health individuals treated with the anticoagulant citrate (BD-Vacutainer tubes) through two rounds of centrifugation. The first round was designed to obtain total plasma and samples were centrifuged at 1,800*× g* for 10 min at room temperature, and the second round was designed to obtain platelet-rich plasma (PRP) and samples were centrifuged at 3,000*× g* for 20 min at room temperature. Then, 20–40 × 10^6^ platelets were stimulated with 10-ng/mL Collagen Type IV in 200 µL of filtered PBS supplemented with 1-mM calcium chloride for 30 min. Platelets were precipitated by centrifugation at 3,000*× g* for 10 min, and sample supernatants were collected. PMPs in the supernatant were washed with DPBS, centrifuged at 16,900*× g* for 60 min, suspended in fresh DPBS, and stored at −70°C until use.

### IC Formation with PMPs (Opsonization)

Platelet-derived MPs generated from three to four healthy donors were thawed, mixed, and quantified by flow cytometry using a similar method as described for MPs from PPP. For opsonization, 8 × 10^5^ PMPs were mixed and incubated with 15 µg/mL purified IgG (isolated from the plasma of 16 SLE-seropositive patients by affinity chromatography) for 60 min at 37°C. The unbound antibodies were removed by washes with 1 mL of PBS and centrifugation at 16,900*× g* for 60 min. The IC formation by PMPs (PMPs-ICs) was assessed by flow cytometry after staining with an *F*(*ab*’)_2_ anti-IgG fragment conjugated with Alexa Fluor 488 for 30 min at 4°C (±20%). PMP mixtures were washed and immediately acquired on a FACS Canto^TM^ II flow cytometer with FACS DIVA software.

### Monocyte Isolation for Culture and Determination of Their Responses

Monocytes were enriched from the peripheral venous blood of patients with SLE and HCs using Rosette Sep, according to the manufacturer’s instructions and a previously published method (31). Approximately 1 × 10^5^ monocytes were cultured alone or in the presence of MPs and MPs-IgG + isolated from patients with SLE that had previously been stained with 2-µM CFSE at a ratio of 1:3 (cells:MPs) to evaluate the binding and uptake of MPs and MPs-IgG + by monocyte subsets. Monocytes were mixed and incubated for 1 h at 37°C in a 5% CO_2_ atmosphere. Next, these cells were stained with antibodies against CD14, CD16, and HLA-DR as described above and analyzed by flow cytometry. Samples were acquired again in the presence of 0.01% v/v trypan blue to quench the fluorescence of bound and non-internalized MPs and estimate the MFI of cells that internalized MPs (31). A similar approach was performed to evaluate the binding and uptake of PMPs and PMPs-IgG + by monocyte subsets, but a ratio of 1:1 (cells:MPs) was used. Some of the binding and uptake experiments using MPs-IgG + and PMPs-IgG + were performed after CD64 on monocytes was blocked with 10 µg/mL of a specific antibody (LEAF Purified antihuman CD64, clone 10.1, Biolegend) for 1 h at 4°C.

Approximately 2.5 × 10^5^ monocytes were cultured alone or with these vesicles at a 1:1 ratio (cells:MPs) in 250 µL of RPMI-1640 supplemented with 5% inactivated and MP-depleted autologous serum (previously centrifuged at 16,900*× g* for 1 h to deplete the remnants MPs after coagulation) for 6 h in sterile polystyrene 12 mm × 75 mm tubes to evaluate monocyte responses to PMPs and PMPs-IgG +. In addition, 10 µg/mL LPS was used as a positive control for monocyte activation in all cultures (data not shown). Supernatants were collected and frozen at −20°C until cytokine levels were assessed using CBA (IL-10, IL-6, IL-8, IL-1β, and TNF-α) and ELISAs (IFN-α, Elabscience, Biotechnology Inc. Houston, TX, USA); monocytes were harvested for an analysis of CD69 and CD64 surface expression using flow cytometry and a similar approach described for monocytes *ex vivo*. At 2 h of culture, monocytes inhibited the expression of CD16, and we analyzed the expression of CD69 and CD64 on the CD14^High^ and CD14^Low^ cells. Some of these experiments were performed after CD64 on monocytes was blocked with 10 µg/mL of a specific antibody for 1 h at 4°C. The concentrations of cytokines in the supernatants were determined using the CBA, according to the manufacturer’s instructions.

### Statistical Analysis

Comparisons among patients with iSLE (*n* = 28) or aSLE (*n* = 32) and the HCs (*n* = 60) and among total patients with SLE (*n* = 60) or RA (*n* = 40) and the HCs (*n* = 60) were performed using the Kruskal–Wallis test and Dunn’s *post hoc* test (data are presented as medians ± interquartile ranges). Comparisons of the MP size and binding and uptake of MPs and MPs-IgG + by monocyte subsets were performed using a two-way ANOVA and the Bonferroni’s *post hoc* test (data are presented as mean ± SD). Statistical significance was set at the critical values of *p* ≤ 0.05, *p ≤ 0.05, **p ≤ 0.01, and ***p ≤ 0.001. Correlations were determined by calculating Spearman’s rank correlation coefficient with a 95% confidence interval. Principal component analysis (PCA) was used to estimate certain interactions between MPs, monocyte subsets, and SLE activity. Analyses were performed by the programs GraphPad Prism version 7.2 (GraphPad Software Inc., San Diego, CA, USA) and Statgraphics Centurion XVI Version 16.1.18 (Statgraphics Corp., Rockville, MD, USA).

## Results

### Platelets are the Main Source of MPs in Patients with Systemic Lupus Erythematosus (SLE)

**Figure 1:**
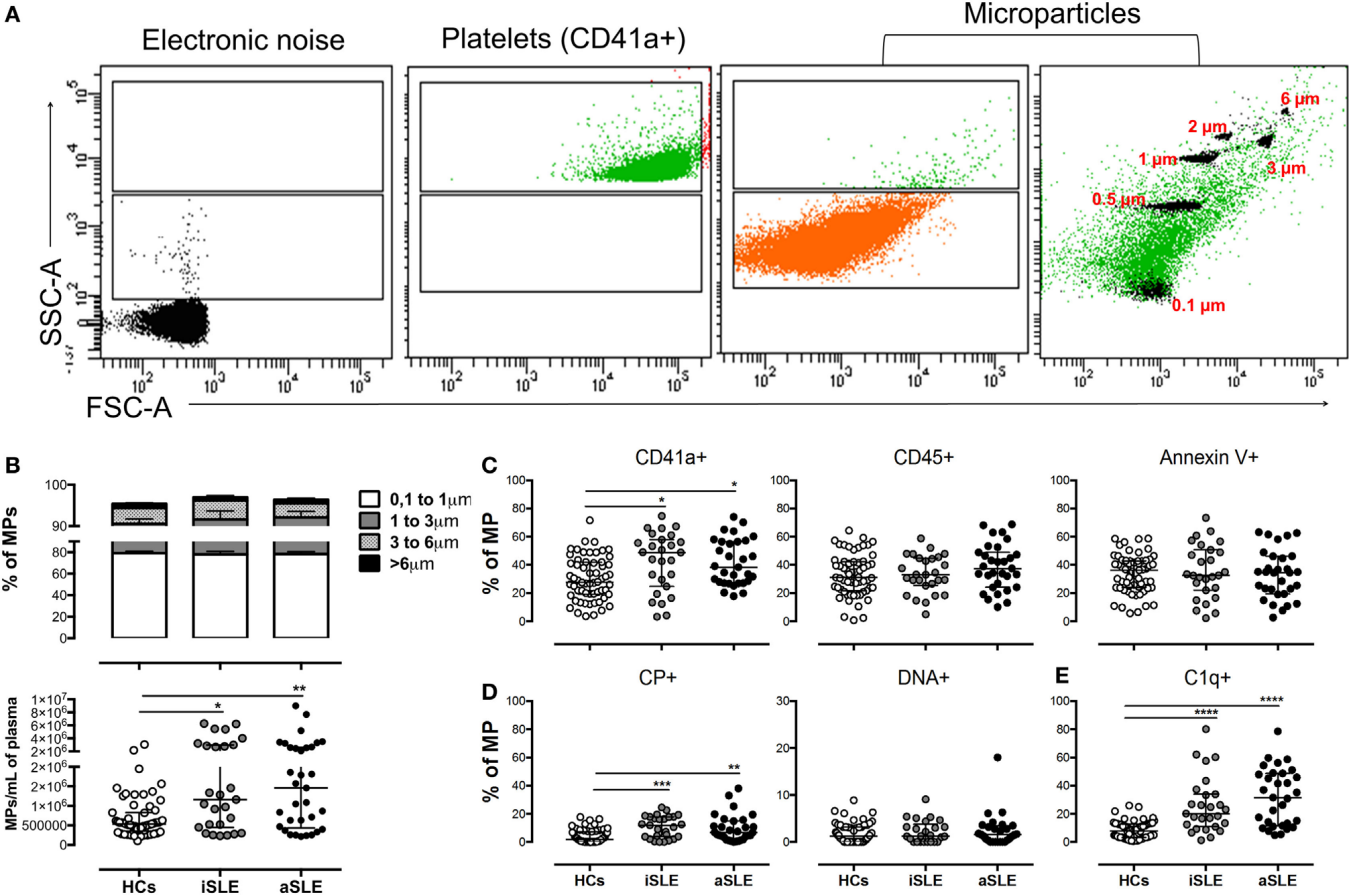
Differences in the number, cell sources, and phenotype of circulating microparticles (MPs) from patients with systemic lupus erythematosus (SLE) and healthy controls (HCs). **(A)** Representative dot plots of the electronic noise, platelets, and MPs. The distribution of MP sizes is shown on the right and the ranking of MP sizes was estimated using standard-sized beads based on FSC-A and SSC-A. The data from a control sample are shown. **(B)** Frequency of MPs of different sizes (upper) and the number (lower) of total MPs in patients with inactive SLE (iSLE) (*n* = 28), patients with active SLE (aSLE) (*n* = 32), and HCs (*n* = 60). Frequency of circulating **(C)** MPs-CD41a +, MPs-CD45 +, MPs-Annexin V +, **(D)** MPs-CP +, MPs-DNA +, and **(E)** MPs-C1q + in patients with iSLE (*n* = 28), patients with aSLE (*n* = 32), and HCs (*n* = 60). Comparisons in MP sizes among the groups were performed using a two-way ANOVA and the Bonferroni’s *post hoc* test. Other comparisons among groups were performed using the Kruskal–Wallis test and Dunn’s *post hoc* test; *p ≤ 0.05, **p ≤ 0.01, and ***p ≤ 0.001.

The strategy used to define the size and granularity of MPs from patients and HCs is shown in Figure 1A. The gate defining MPs was located above the electronic noise and below the platelets (Figure 1A). In addition, different controls were included and performed during the standardization phase of MP detection to ensure a minimal contribution of protein aggregates, the vesicular nature of these structures, the absence of contamination with smaller vesicles such as exosomes (by size exclusion and CD63 expression), and the concentration of MPs used in the staining and acquisition procedures (Figure S1 in Supplementary Material). The presence of actin in these structures was also observed (data not shown –DNS–). Therefore, we focused our study on these extracellular vesicles, which display phenotypic characteristics that correspond to MPs by flow cytometry and hence will be called MPs hereafter. The size of 80% of the MPs ranged from 0.1 to 1 µm, and their sizes did not differ among the study groups (Figure 1B). The circulating MP count was significantly higher in patients with iSLE (*p* < 0.01) and patients with aSLE (*p* < 0.001) compared with HCs (Figures 1A,B) and significantly higher in total patients with SLE compared with patients with other systemic autoimmune diseases (SADs), such as RA (Figure S2A in Supplementary Material). Patients had higher percentages of MPs-CD41a + than did HCs, regardless of disease activity (Figure 1C). Low proportions of MPs positive for endothelial (CD105 +) and erythroid (CD235a +) markers (Figure S3A in Supplementary Material) and negative for all linage markers evaluated were observed in the study groups (DNS). Leukocytes were an important source of MPs (Figure 1C), and similar proportions of MPs positive for CD3, CD19, CD33, CD16, and CD14 as well as MPs-Annexin-V + were observed between patients and HCs (Figure 1 C; Figure S3A in Supplementary Material). Patients with either iSLE or aSLE showed a higher proportion of MPs positive for citrullinated peptides (CP +) than HCs (Figure 1D), and an enrichment of CP + on MPs-CD41a + was observed (Figure S3B in Supplementary Material). No differences in the proportions of MPs-DNA + (Figure 1D) and MPs-RNA + (DNS) were observed among the study groups.

### MPs from Patients with SLE Form ICs with IgM and IgG

**Figure 2:**
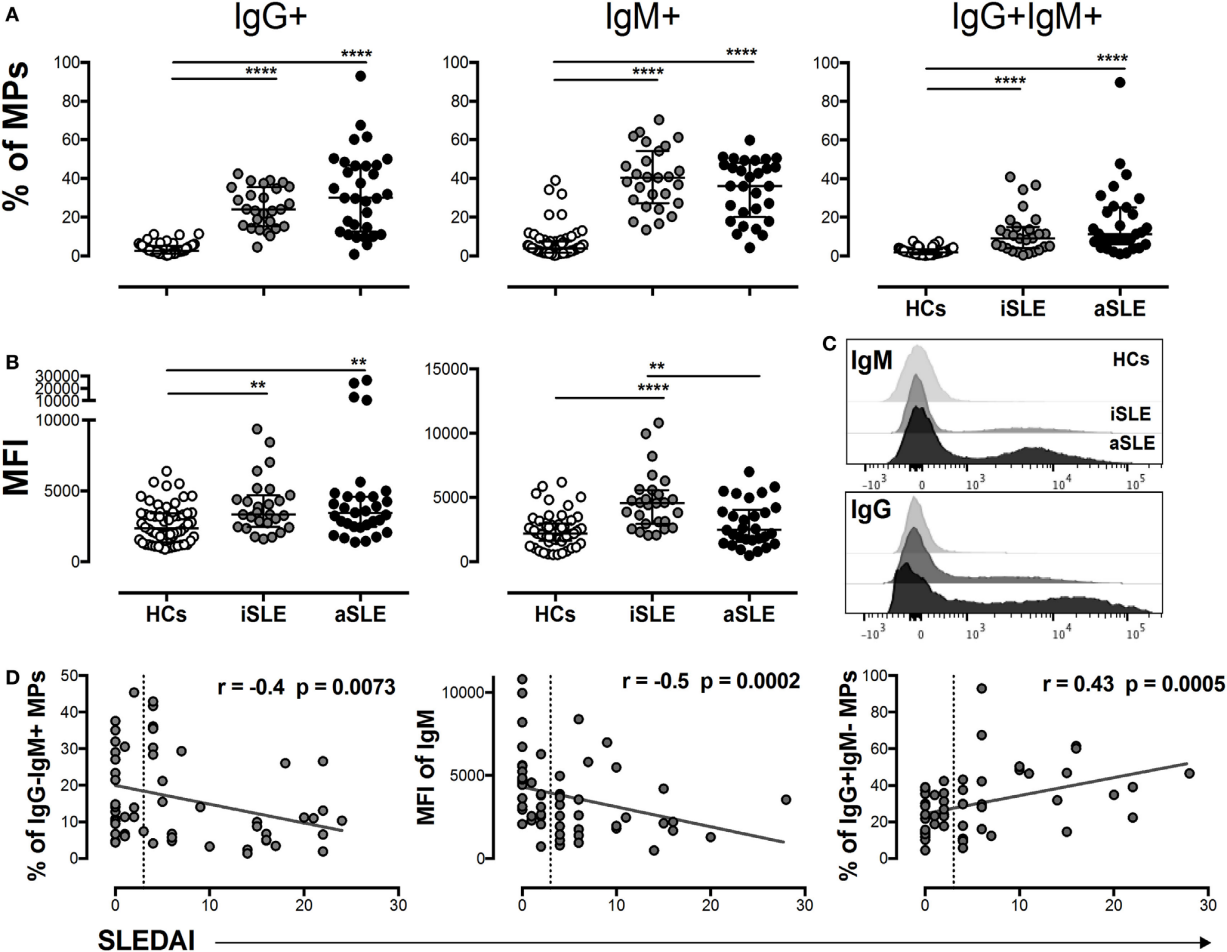
MPs-IgG + are associated with systemic lupus erythematosus (SLE) activity. **(A)** Frequency of circulating MPs-IgG + IgM−, MPs-IgG − IgM +, and MPs-IgG + IgM + in patients with inactive SLE (iSLE) (*n* = 28), patients with active SLE (aSLE) (*n* = 32), and healthy controls (HCs) (*n* = 60). **(B)** MFIs of IgG and IgM on circulating microparticles (MPs). **(C)** Representative histograms show IgM (upper) and IgG (lower) expression on MPs from patients with aSLE (black), patients with iSLE (dark gray), and HCs (light gray). **(D)** Analysis of the correlations between the MPs-IgG−IgM +, MPs-IgM + MFI, and MPs-IgG + IgM− frequencies and systemic lupus erythematosus disease activity index (SLEDAI) scores. Comparisons among groups were performed using the Kruskal–Wallis test and Dunn’s *post hoc* test; **p ≤ 0.01 and ***p ≤ 0.001. Correlation analyses were performed by determining Spearman’s rank correlation coefficients and 95% confidence intervals; the dotted line indicates the cut-off point between patients with iSLE (SLEDAI < 4) and those with aSLE (SLEDAI ≥ 4).

A significantly higher MPs-C1q + frequency was observed in patients with SLE than that observed in HCs (Figure 1E), suggesting that MPs formed ICs in these individuals. Therefore, the presence of IgG and IgM on these vesicles was evaluated. Patients had a higher frequency of MPs-IgG + IgM−, MPs-IgG-IgM +, and MPs-IgG + IgM + compared with HCs (Figure 2A). In addition, the MFI of IgG on MPs from patients was significantly increased compared with the HCs (Figures 2B,C), independent of disease activity. In contrast, a higher MFI of IgM was observed on MPs from patients with iSLE compared with patients with aSLE and HCs (Figures 2B,C). The sources of MPs-C1q +, MPs-IgG + IgM−, and MPs-IgG-IgM + were leukocytes and platelets in patients with SLE (Figure S3B in Supplementary Material). The proportion of MPs-IgG-IgM + and the MFI of IgM on MPs was negatively correlated with SLEDAI scores, whereas the frequency of MPs-IgG + IgM− was positively correlated with disease activity (Figure 2D). Total levels of circulating ICs-IgG + evaluated using an ELISA were increased in patients with SLE compared with HCs and were positively correlated with SLEDAI scores and MPs-IgG + IgM− levels evaluated by flow cytometry. Indeed, when MPs from plasma samples from patients were separated and evaluated by ELISA, most of the total circulating ICs-IgG + were located on these vesicles (Figures S4A,B in Supplementary Material). Patients with aSLE exhibited a higher concentration of serum anti-C1q antibodies, and this increase was positively associated with SLEDAI scores (Figure S4C in Supplementary Material); however, the increase in the levels of this factor and the frequency of MPs-C1q + was not correlated (Figure S4C in Supplementary Material). The presence of MP-forming ICs was also evaluated in patients with other SADs, such as RA, and similar to patients with SLE, patients with RA had a higher frequency of MPs-IgG + than did HCs, although the value was significantly lower than that for patients with SLE (Figure S2A in Supplementary Material).

### Numbers of Non-Classical Monocytes Negatively Correlated with Systemic Lupus Erythematosus Disease Activity Index Scores

**Figure 3:**
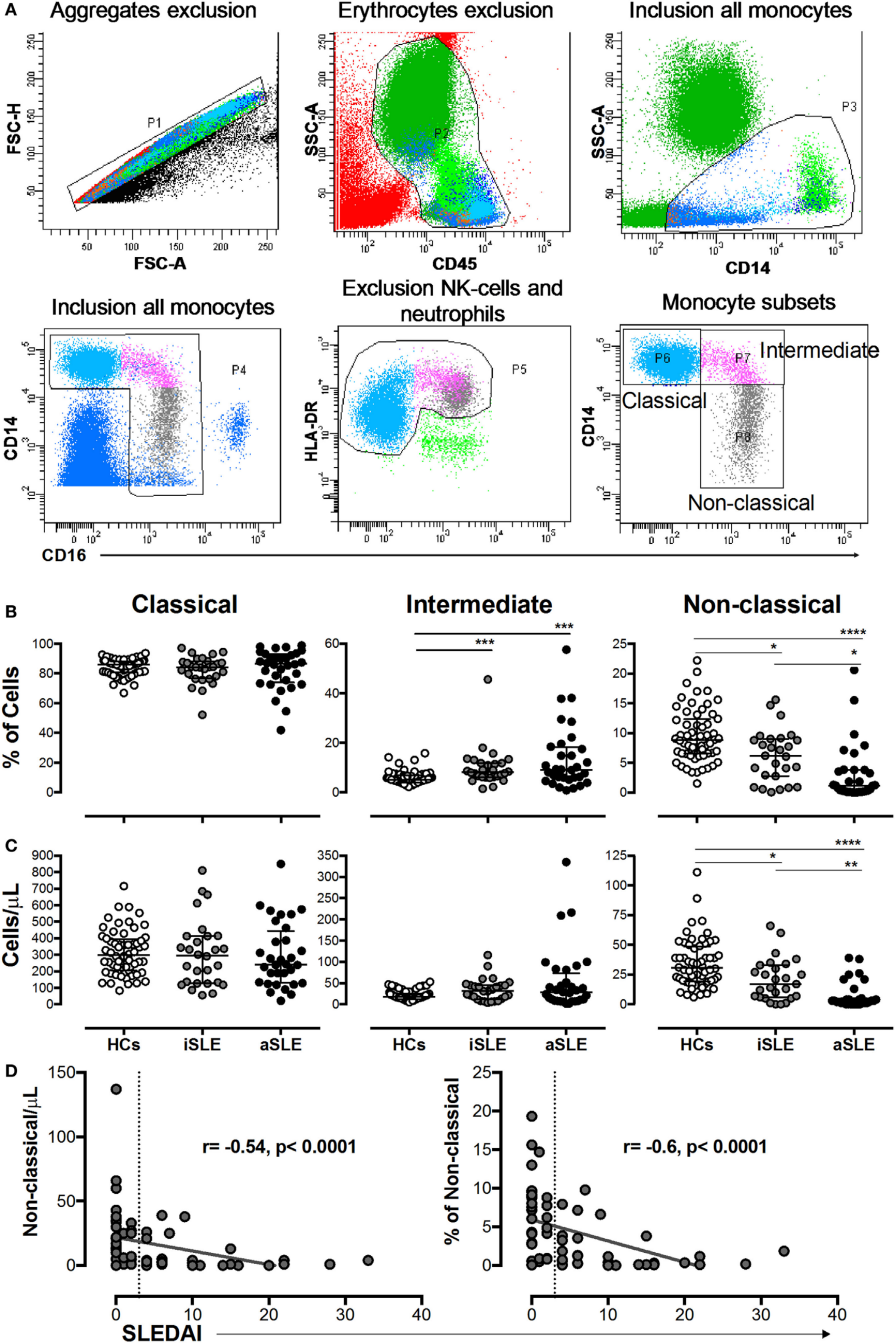
The percentage and number of non-classical monocytes were decreased in patients with systemic lupus erythematosus (SLE) and correlated with systemic lupus erythematosus disease activity index (SLEDAI) scores. **(A)** Gating strategy used to determine monocyte subsets. **(B)** Frequencies and **(C)** absolute numbers of classical, intermediate, and non-classical monocytes in patients with inactive SLE (iSLE) (*n* = 28), patients with active SLE (aSLE) (*n* = 32), and HCs (*n* = 60). **(D)** Analysis of correlations between the absolute number and frequency of non-classical monocytes with SLEDAI scores. The dotted line indicates the cutoff point between patients with iSLE (SLEDAI < 4) and those with aSLE (SLEDAI ≥ 4). Comparisons among the groups were performed using the Kruskal–Wallis test and Dunn’s *post hoc test*: *p ≤ 0.05, **p ≤ 0.01, and ***p ≤ 0.001. Correlation analyses were performed by calculating Spearman’s rank correlation coefficients and 95% confidence intervals.

The gating strategy used to define monocyte subsets is shown in Figure 3A. A significantly higher frequency of intermediate monocytes was observed in patients with SLE compared with HCs (Figure 3B). The proportion and number of non-classical monocytes was significantly decreased in patients with SLE compared with HCs and in patients with aSLE compared with patients with iSLE (Figures 3B,C). No changes were observed in the numbers of classical monocytes among the studied groups (Figures 3B,C). Significant and negative correlations were observed between the percentage and number of non-classical monocytes with SLEDAI scores (Figure 3D).

### Altered Expression of Receptors Associated with MP Recognition on Monocytes from Patients with SLE

**Figure 4:**
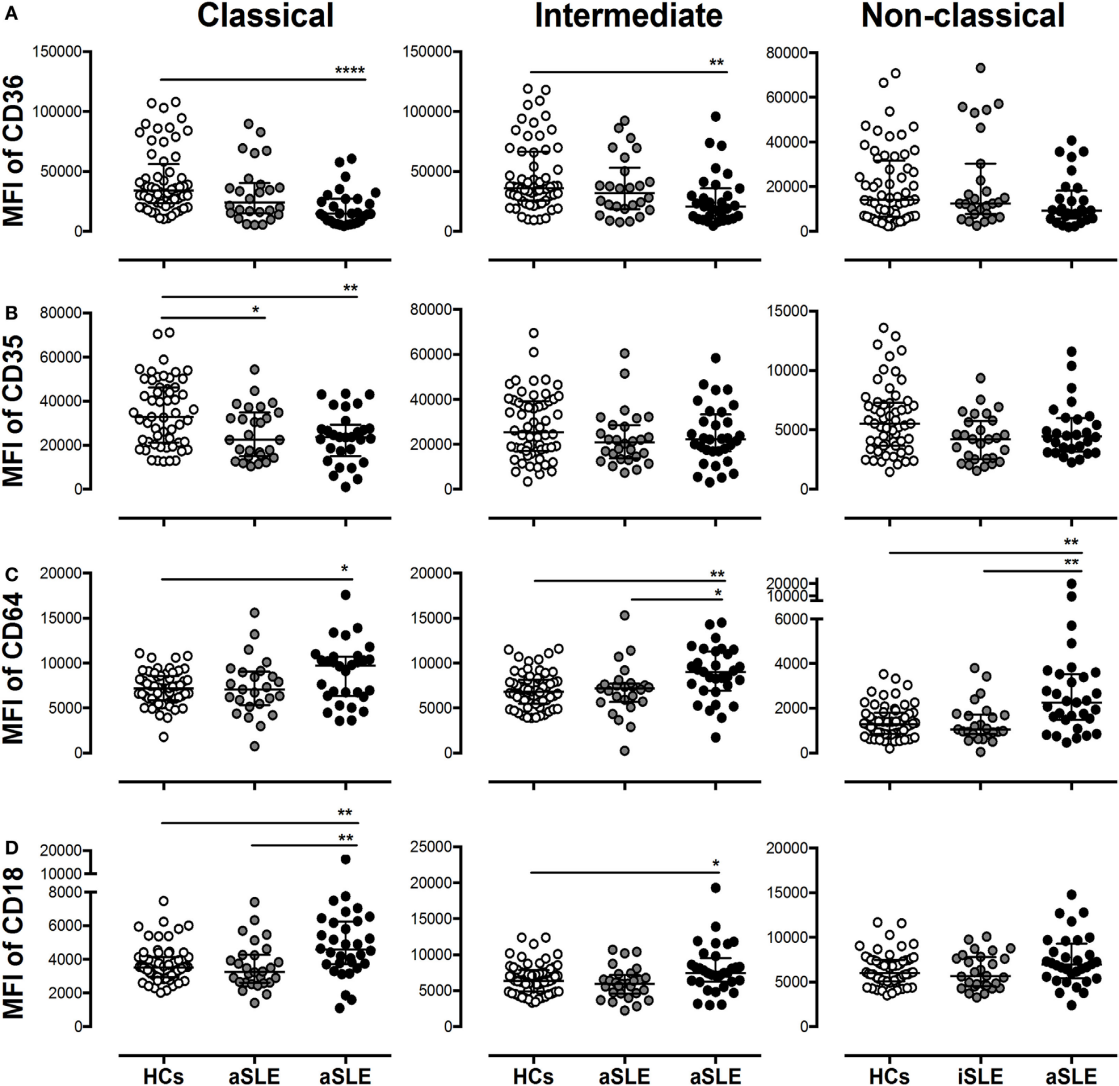
Subsets of monocytes from patients with systemic lupus erythematosus (SLE) display alterations in the expression of putative microparticle (MP) receptors. The MFIs of **(A)** CD36, **(B)** CD35, **(C)** CD64, and **(D)** CD18 on classical, intermediate, and non-classical monocytes from patients with inactive SLE (iSLE) (*n* = 28), patients with active SLE (aSLE) (*n* = 32), and healthy controls (HCs) (*n* = 60). Comparisons among groups were performed using the Kruskal–Wallis test and Dunn’s *post hoc* test; *p ≤ 0.05, **p ≤ 0.01, and ***p ≤ 0.001.

CD36 expression was reduced in classical and intermediate monocytes from patients with aSLE compared with HCs (Figure 4A). The expression of this receptor was not altered in the monocyte subsets from patients with other SADs (such as RA) compared with HCs (Figure S2C in Supplementary Material). No differences in the MFIs of the C1q receptor CD93, FcγRII (CD32), and FcμR were observed in monocyte subpopulations from patients compared with HCs (DNS). The expression of the C1q receptor CD35 was reduced in classical monocytes from patients with SLE (Figure 4B). The MFI of FcγRI (CD64) increased in all monocyte subpopulations from patients with aSLE compared with HCs and in intermediate and non-classical monocytes from patients with aSLE compared with patients with iSLE (Figure 4C). In addition, CD64 expression was increased in all monocyte subsets from total patients with SLE compared with patients with other SADs, such as RA (Figure S2B in Supplementary Material). FcγRIII CD16 expression was decreased in intermediate and non-classical monocytes from patients with aSLE compared with patients with iSLE and HCs (DNS).

The evaluation of different integrins (CD11a, CD11b, CD11c, and CD18) and complement receptors (CRs) (CR3 and CR4) revealed only a significant increase in the MFI of CD18 on classical monocytes from patients with aSLE compared with patients with iSLE and HCs, and on intermediate monocytes from patients with aSLE compared with HCs (Figure 4D and DNS).

### Association of MPs and Mononuclear Phagocytes with SLE Activity

**Figure 5:**
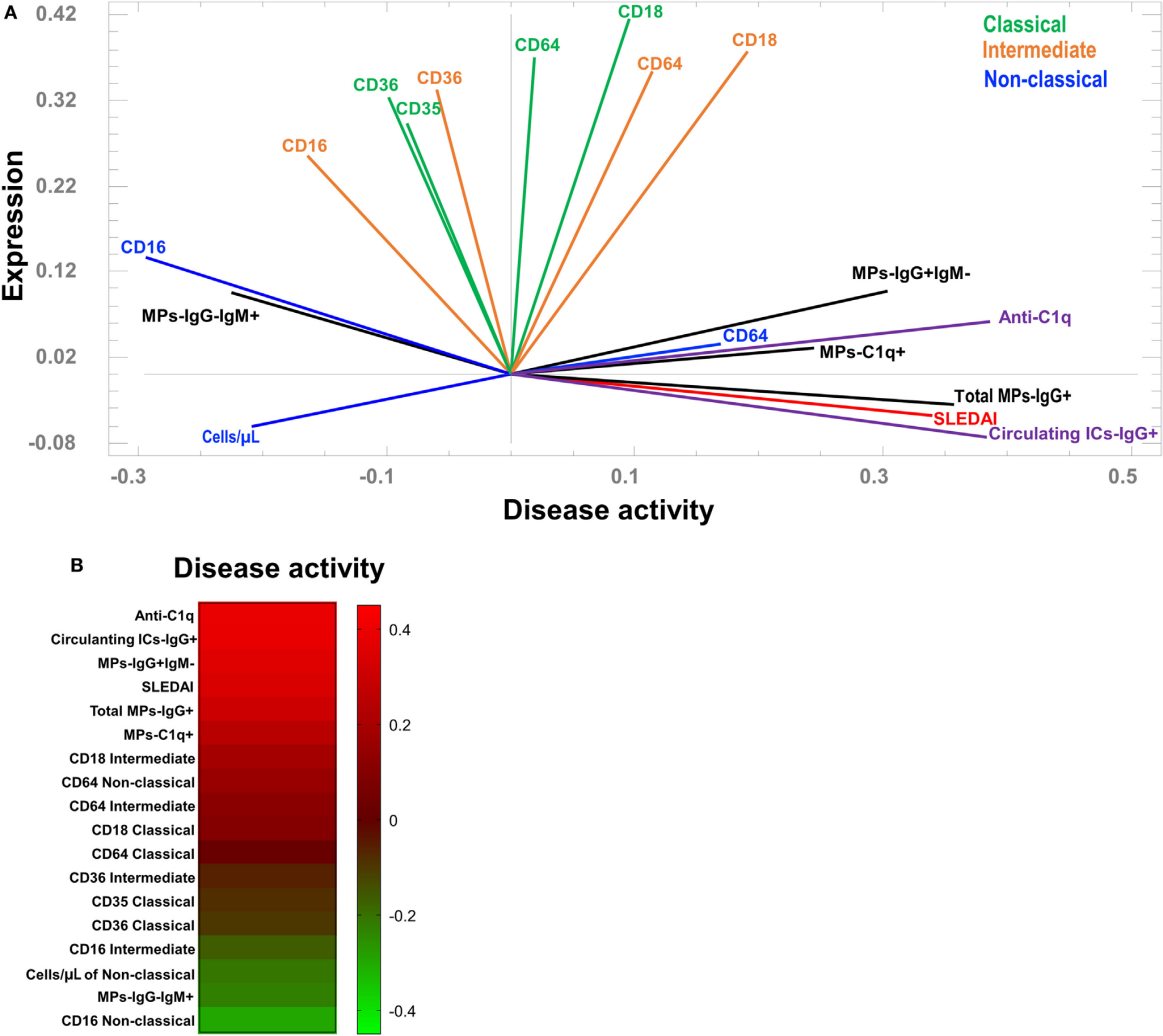
Association between the phenotypes of microparticles (MPs) and monocyte subsets in patients with systemic lupus erythematosus (SLE). **(A)** Principal component analysis showing the correlations between the phenotypes of monocyte subpopulations and MPs with systemic lupus erythematosus disease activity index (SLEDAI) scores. **(B)** Heat map of the weights of each variable in component 1 (disease activity).

A PCA was performed to define associations among the main phenotypic changes observed in monocyte subsets and MPs with disease activity. Two components explain 48% of the variability in the system; component 1 was related to disease activity and component 2 was related to the expression of the parameters measured in the present study. Three main groups were clearly observed, based on their eigenvalues. The first included patients with the highest SLEDAI scores and was closely related to the percentage of total MPs-IgG + (including all MPs that were positive for IgG independent of IgM expression) and the concentration of total circulating ICs-IgG +. In the same group, which was not as narrowly associated with higher SLEDAI scores, correlations with other variables were observed, including the MFI of CD64 in non-classical monocytes, MPs-C1q +, MPs-IgG + IgM−, and serum levels of anti-C1q (Figure 5A). In contrast to the first group, particularly regarding the higher SLEDAI scores, the second group was mainly defined by the MFI of CD16 in non-classical monocytes and MPs-IgG-IgM +. In addition, the number of non-classical monocytes was correlated with this group (Figure 5A). A third group lying between these two groups was observed and had a wide spectrum of different phenotypic markers on intermediate and classical monocytes (Figure 5A). A heat map was constructed to visualize the weight of each variable in the two components described. This approach corroborated that total MPs-IgG +, MPs-IgG + IgM−, and CD64 expression on non-classical monocytes were positively correlated with disease activity (Figure 5B). In contrast, MPs-IgG-IgM +, the increase in CD16 expression, and the number of non-classical monocytes were negatively correlated with disease activity (Figure 5B).

### Binding and Uptake of MPs-IgG + by Classical and Intermediate Monocytes, and PMPs-IgG + Inducing Monocyte Activation

**Figure 6:**
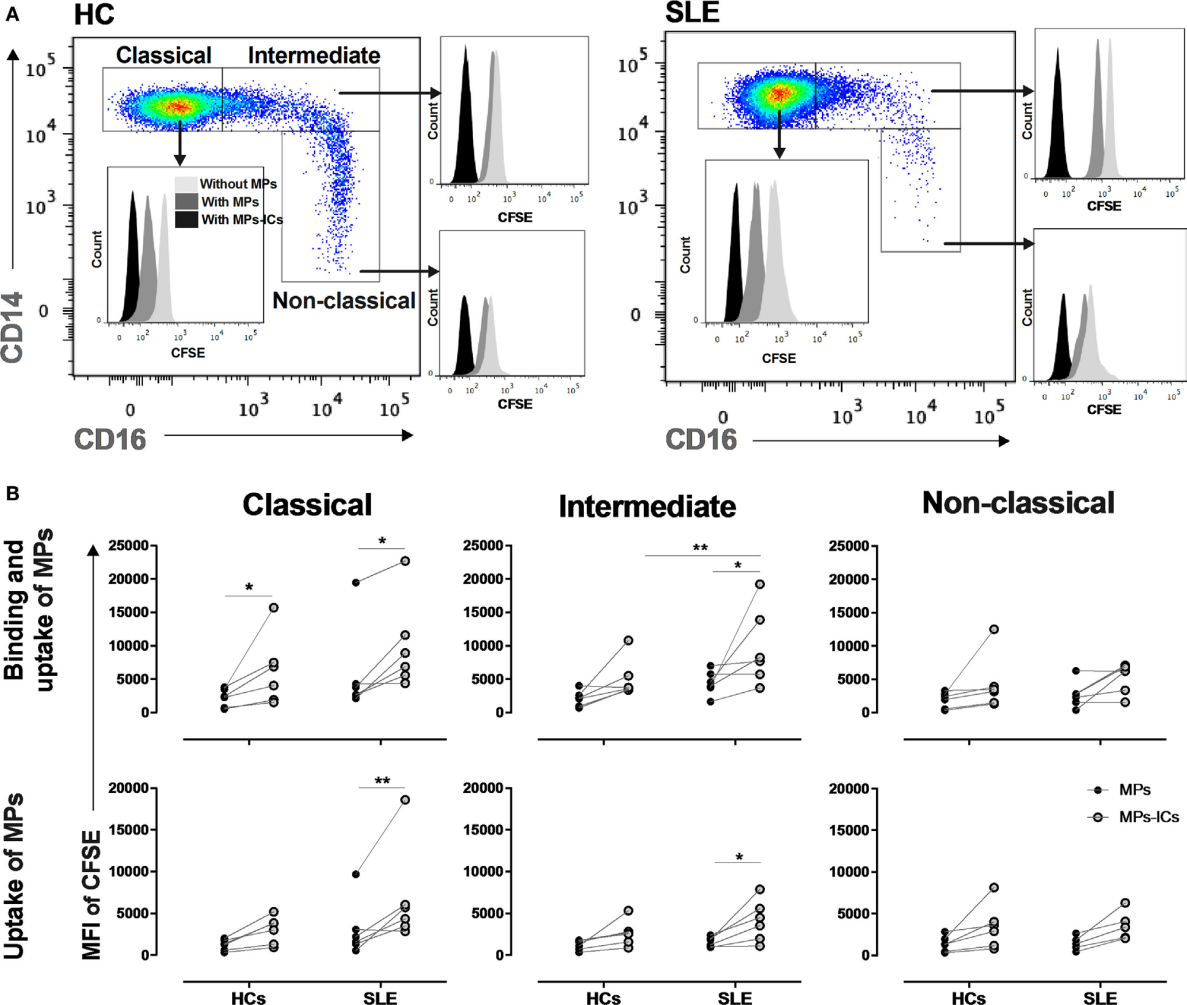

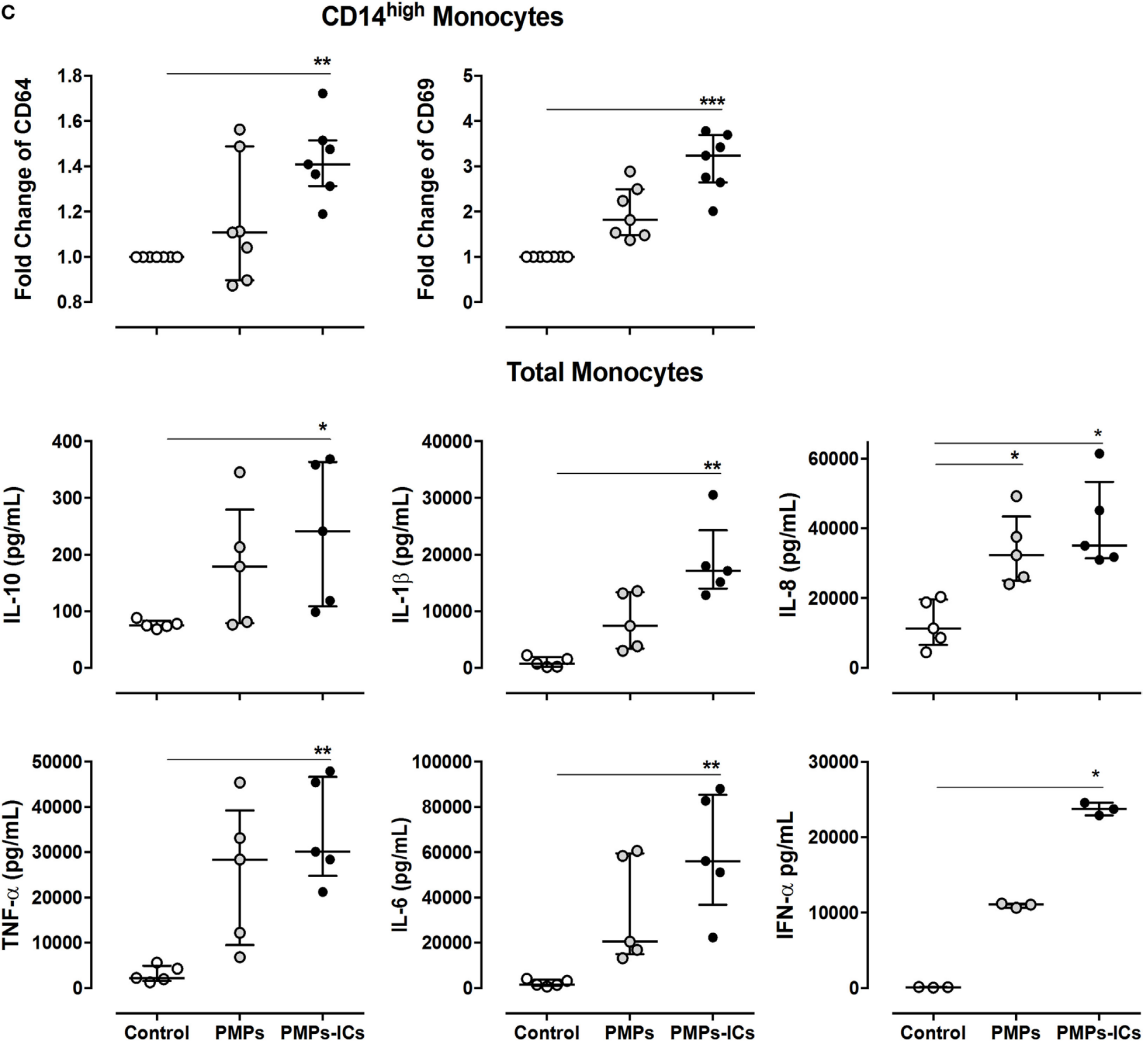
Monocyte subsets showing the binding and uptake of MPs-IgG +, and PMPs-IgG + inducing monocyte activation. **(A)** Representative image showing the binding and uptake of microparticles (MPs) and MPs-ICs (3:1 vesicle:cell ratio) by monocyte subsets in healthy individuals (left panel) and patients with systemic lupus erythematosus (SLE) (right panel). **(B)** Binding and uptake (upper panel) and uptake (lower panel) of MPs and MPs-ICs (3:1 vesicle:cell ratio) by classical, intermediate, and non-classical monocytes from HCs and SLE patients. Comparisons between the groups were performed using the Wilcoxon signed-rank test; *p ≤ 0.05, **p ≤ 0.01, and ***p ≤ 0.001. **(C)** CD64 and CD69 expression in CD14^High^ monocytes (upper panel) and cytokine levels in supernatants of monocytes stimulated with PMPs and PMPs-ICs (lower panel). Comparisons among the groups were performed using the Kruskal–Wallis test and Dunn’s *post hoc* test; *p ≤ 0.05, **p ≤ 0.01, and ***p ≤ 0.001.

Circulating MPs isolated from patients with SLE were labeled with CFSE and cultured with total monocytes to evaluate whether monocyte subsets bind and take up these vesicles. Controls for the quenching efficiency of CFSE-labeled MPs and MPs-ICs are shown in Figure S5A in Supplementary Material. Classical and intermediate monocytes from patients with SLE bound and took up more MPs-IgG + than MPs, whereas these findings were only observed for classical monocytes in HCs (Figures 6A,B). In addition, intermediate monocytes from patients with SLE bound and took up higher levels of MPs-IgG + than HCs. In non-classical cells, significant differences were not observed, although an increasing trend was noticed (Figures 6A,B). CD64 blockade had no effect on the binding and uptake of MPs-ICs by monocyte subsets (Figures S5B,C in Supplementary Material).

Since PMPs were the most frequent type of MPs detected in patients with SLE, we generated these structures from enriched platelets obtained from HCs and opsonized them with or without IgG from patients with SLE. The results from the *in vitro* generation of PMPs and their opsonization are shown in Figures S6A,B in Supplementary Material. The patterns of binding and uptake of these PMPs and PMPs-IgG + were similar in HC monocyte subsets compared with circulating vesicles from patients with SLE, indicating that more PMPs-ICs were bound and taken up than were non-opsonized PMPs (Figure S6C in Supplementary Material). Controls for the quenching efficiency of CFSE-labeled PMPs-ICs are shown in Figure S6D in Supplementary Material. CD64 blockade had no effect on the binding and uptake of PMPs-ICs by monocyte subsets (Figure S6E in Supplementary Material).

Monocytes from HCs were cultured with *in vitro* generated PMPs and PMPs-IgG + to determine whether MPs-IgG + have any direct effect on these cells. PMPs-IgG + significantly increased the expression of CD64 and CD69 in CD14^High^ monocytes (Figure 6C). No differences were observed in CD14^Low^ monocytes (DNS). PMPs-IgG + significantly increased the accumulation of IL-10, IL-1β, IL-8, TNF-α, IL-6, and IFN-α (Figure 6C). These responses were not observed in monocytes cultured with IgG alone (Figure S6F in Supplementary Material and DNS), and CD64 blockade had no effect on the PMPs-IgG-induced accumulation of IL-1β, TNF-α, IL-6, and IL-10 in monocyte supernatants (Figure S6F in Supplementary Material). Based on these results, opsonized MPs have a direct effect on monocytes, inducing their activation.

## Discussion

Here, MPs and monocyte subset phenotypes were associated with disease activity in patients with SLE. On one hand, a high number of and the MFI of CD16 on non-classical monocytes and an increased MPs-IgG-IgM + frequency were negatively correlated with SLEDAI scores. A high CD64 MFI in non-classical monocytes, increased percentages of MPs-IgG + IgM−, total MPs-IgG +, and MPs-C1q +, and increased levels of total circulating IC-IgG + and anti-C1q were observed in patients with aSLE. Based on this evidence, both monocytes and MPs may interact *in vivo* to induce different aspects of SLE activity.

Patients with SLE displayed an elevated number of total circulating MPs, mainly derived from platelets. This finding is consistent with previous studies reporting that 2–10 times more MPs in the blood from patients with SLE compared with HCs (38). MPs in patients with SLE are mainly derived from platelets (PMPs) (14, 39, 40). However, other authors have reported a decrease in the number of plasma MPs in patients with SLE compared with that in HCs (41). This finding does not agree with our results, possibly because we evaluated freshly isolated samples instead frozen PPP. Although freezing procedures have not been shown to alter MPs (42), in our study, this conclusion was accurate for the surface expression of different proteins but not for the counts and the detection of ICs formed from these structures (DNS). In addition, the increase in the MP counts in our cohort was not related to any of the clinical variables evaluated, such as renal, hematological, neurological, immunological, cardiovascular, skeletal muscle, and mucocutaneous involvement, or even with different doses of glucocorticoids (DNS). Previously, an increase in the number of circulating endothelium-derived MPs in patients with SLE has been reported (21, 41). However, the linage markers used for this analysis were CD31 and CD146 instead CD105 that was used in our case. All these molecules are linage markers accepted for the characterization of endothelial cells; however, all of them can be expressed in different proportion on other cells like leukocytes and platelets (43–47). This may be an explanation for the discrepancy between these reports and our findings.

Researchers have not conclusively determined the mechanism by which PMP levels are increased in patients with SLE; however, PMP levels do not appear to be related to the disease activity, as no differences were observed in SLEDAI scores. Nevertheless, it may be a consequence of platelet activation, such as by autoantibodies against the platelet glycoproteins (48) and lupus anticoagulant (49), or through inflammatory mediators such as TNF-α and IL-1β, which induce platelet activation. Indeed, patients with SLE present different inflammatory changes associated with massive fibrin networks and micro-thrombus formation. Thus, platelets can generate PMPs when participating in these responses (50). Notably, although the mechanism remains to be defined in SLE, platelets have been shown to bind and activate monocytes through serotonin release (51). Therefore, we propose that PMPs induce similar responses in monocytes from patients with SLE, mainly when PMPs are forming ICs. This hypothesis was supported by the PCA results and partially corroborated with the findings from *in vitro* cultures, in which we observed that MPs-IgG + were bound and internalized by classical and intermediate monocytes from patients with a greater extent than MPs, and PMPs-ICs induced the expression of CD69 and CD64 and the release of different cytokines by monocytes.

Although platelets lack a nucleus, they can contain different autoantigens that enable them to form ICs, such as functional mitochondria containing cardiolipin and mitochondrial DNA (mtDNA). These highly inflammatory organelles are released from platelets and have been detected outside cells and free in the circulation of patients with various pathologies, including those with SLE (52). In contrast, another explanation for the observation of platelets containing autoantigens in patients with SLE is the high levels of nucleosomes that have been detected in the blood of patients with several inflammatory conditions, such as autoimmune diseases (53). Histones are cationic proteins that associate with DNA in nucleosomes, and Fuchs et al. showed that histones bind to platelets, leading to their aggregation, calcium flux, and shape changes (54); histones H4 and H3 are the main histones responsible for activating human platelets by binding to TLR2 and TLR4 (55). Therefore, we postulate that this phenomenon leads to the release of MPs with nucleosomes attached to their surface from platelets, which in turn favors IC formation. However, since we did not evaluate which autoantigens on PMPs or autoantibodies were responsible for IC formation, further studies are required to resolve this issue.

As observed in our results, opsonization does not seem to occur in all PMPs since only approximately 10–25% of these vesicles formed ICs *in vitro* with the IgG from patients with SLE; instead *in vivo*, more than 30% of PMPs were opsonized in a considerable number of patients. Thus, for some reason, PMPs from patients with SLE express more autoantigens *in vivo* than PMPs obtained from the *in vitro* stimulation of platelets from HCs. However, platelet activation appears to be important for the expression of those autoantigens since unstimulated platelets generated PMPs but were not opsonized *in vitro*. Therefore, a greater level of membrane exposure of autoantigens on PMPs must occur in patients with SLE, which allows the recognition of more IgG autoantibodies. This hypothesis partially explains the observed lower binding and uptake of CFSE-labeled PMPs-IgG + generated *in vitro* than MPs-IgG + obtained from patients.

The physiological role of MPs may be altered in patients with SLE, mainly due to variations in their cell source and contents observed in the present and other studies (4, 56). The cell death and activation of a variety of cells in patients with SLE (57) as well as defects in vesicle clearance (31) may favor MP accumulation and allow the formation of potentially pathogenic modifications and ICs. The presence of CP in MPs suggested that these vesicles were modified in the extracellular milieu or that they were derived from sources rich in those polypeptides. The participation of MPs-CP + in the pathogenesis of SLE remains to be defined; however, CP is recognized by TLR4 (58). Notably, the serum concentrations of anti-CCP autoantibodies were similar in patients with SLE and HCs (DNS); therefore, MPs-CP + likely function more as alarmins than as autoantigens in patients with SLE.

Since nucleic acids are extensively involved in the pathogenesis of SLE, the presence of these molecules on the surface of MPs was also analyzed in the present study. However, we did not observe differences in the frequency of MPs-DNA + and MPs-RNA + from patients with SLE compared with HCs. Importantly, PI only stains nucleic acids on the surface of intact MPs; therefore, based on these results, we could not conclusively determine the total amount of DNA or RNA inside MPs or the total frequency of positive MPs to nucleic acids (inside and outside) in patients with SLE. Although PI is able to bind to relatively few base pairs (from 4 to 5), we may not have detected any differences because a permeabilization step was not included in our protocol to avoid any alteration in the MPs structure. Thus, we cannot ignore that this technical approach introduced a bias to characterize only surface DNA and RNA on MPs of this study. Therefore, other approaches are needed for the detection of differences in nucleic acids such as the use of anti-DNA and anti-chromatin antibodies, PCR, southern blot, northern blot, and image cytometry. However, regarding the use of antibodies is important to notice that, although anti-DNA antibodies have been shown to bind to the MP surface (17), those antibodies also react with other cell components. For example, Sisarak et al. identified MPs-DNA + based on the binding of the PR1-3 antibody to these vesicles; however, this antibody is specific for anti-DNA/histone H2a/H2b (5). In addition, anti-DNA antibodies bind to nucleosomes (17) and cell membranes (59, 60). Furthermore, anti-DNA antibodies are also able to simultaneously bind the DNA and the proteins attached to the MP surface (59, 60). Therefore, in patients with SLE, these antibodies recognize the DNA (or RNA)-protein complex, and thus, instead of the frequency of MPs-DNA + or MPs-RNA +, evaluations of the type of complexes (nucleic acids with nucleosomes or ribonucleoproteins) present in MPs from patients with SLE may be more relevant and informative. However, this speculation requires further investigation in our patients.

Other authors have reported a reduced proportion of MPs-Annexin-V + in patients with SLE (13), which contradicts our findings. Furthermore, in the present study, monocyte subsets expressed lower levels of CD36. Thus, although MPs from patients with SLE express PS, monocytes do not remove these vesicles efficiently or silently (61). These findings also partially explain the larger number of circulating MPs observed in patients SLE, as previously suggested for ACs in patients with this disease (57). However, additional studies are required to evaluate these assumptions since we did not observe differences in MP internalization between monocytes from patients and HCs.

Microparticles from patients with SLE form ICs that are associated with complement activation and disease activity (13, 62). However, the present study is the first report showing that MPs-IgG +, but not MPs-IgM +, were positively correlated with disease activity. These results, together with the increased uptake of MPs-CIs by monocytes from patients with SLE compared with HCs, independent of CD64, suggest that MPs-IgG + preferentially interacts with monocytes through other FcγR, possibly enabling the pro-inflammatory activation of these cells (63). The expression of CD64 on monocyte surface is considered a biomarker of SLE activity (64). *In vitro*, type-I IFNs (IFNs-I) and stimulation with some TLR agonists induce CD64 expression on monocytes (63). Here, PMPs-CIs induced CD64 and CD69 expression and IFN-α production, indicating that these structures directly activated monocytes.

Type-I IFN overproduction is a hallmark of SLE, and these factors are mainly produced by plasmacytoid DCs (pDCs), monocytes, and macrophages (65). The production of these cytokines is induced by endosomal TLRs, such as TLR3, TLR7, and TLR9, and by some surface TLRs, such as TLR4 (66). It has been observed that ICs containing DNA and RNA induce the production of IFNs-I by monocytes of healthy individuals *in vitro* (67). Although we did not observe more MPs-DNA + and MPs-RNA + frequency in SLE patients, our results showed that PMPs-IC induce IFNs-I secretion, possible through the binding of FcγR and TLR on the plasma membrane and once internalized, these vesicles can exposure the nucleic acids that contain to interact with endosomal TLRs. IFN-α has been positively correlated with the increase in CD64 expression in monocytes from patients with SLE (64). Mononuclear cells threated with agonists of TLR7 and TLR9 increased the expression of CD64 in monocytes of healthy individuals; likewise, the exposure of these cells to sera from patients with SLE positively regulated transcription and surface expression of CD64 in a manner dependent on the concentration of this cytokine (63). Therefore, MPs-ICs seem to contribute to the activation of monocytes and the IFN-I hallmark in SLE patients.

The presence of C1q on MPs also suggested that MPs-ICs induce classical complement activation. According to the PCA, MPs-C1q + appear to have a role in SLE activity. The binding of free C1q to monocytes negatively regulates their inflammatory response induced by HMGB1 (68). Therefore, MPs-IgG + may be a potential target for C1q consumption, avoiding this regulatory pathway. The recognition of C1q-opsonized ICs through putative C1q receptors (CD35 and CD93) is another pathway for the non-inflammatory removal of ACs (69), producing IL-10 (27) and avoiding the release of alarmins (70); this pathway is also postulated for MPs. However, we detected reduced CD35 expression in patients with iSLE and an even more significant reduction in patients with aSLE. Thus, similar to CD36, the internalization of MPs through this pathway may not be efficient in these patients, favoring their accumulation and subsequent recognition by anti-C1q antibodies. These changes would alter the silent removal of MPs and activate the complement cascade.

The presence of the complement fractions C3b and C4b on MPs has been reported previously (71), indicating that these vesicles are potentially recognized by the CR3, formed by integrins αM and β2 (CD18). According to Wolf et al., CR3 increases the expression of IL-6 and CCL2 in macrophages (72). Since we observed increased CD18 expression in the classical and intermediate monocytes from patients with aSLE, this pathway may represent another mechanism for the MP-mediated inflammatory activation of monocytes.

In contrast to MPs-IgG + IgM−, the MP-IgG-IgM + frequency was negatively correlated with SLE activity. Poly-reactive IgM-natural autoantibodies have been shown to bind self-reactive IgG, providing a mechanism to protect the host from high-affinity autoantibodies (73, 74). IgM autoantibodies that bind to neo-epitopes on ACs are significantly more plentiful in patients with iSLE than in patients with aSLE and are associated with less-severe organ damage (75). Furthermore, IgM antibodies promote the non-inflammatory clearance of MPs by monocytes and macrophages more effectively than ACs (76, 77), which may occur through FcμR on monocytes (78). Therefore, IgM may facilitate the silent removal of MPs and avoid the pathogenic effect of ICs-IgG + on monocytes from patients with SLE. This mechanism of MP removal may negatively regulate monocyte activation in patients with iSLE when other pathways, such as those mediated by CD36 and CD35, are activated to a lower extent.

Non-classical monocytes patrol the endothelium of microvasculature, removing ACs, ICs, and MPs (79). In the present study, the decrease in the number of these cells in patients with SLE was positively correlated with disease activity. In our previous study, we observed the same reduction in another group of patients with SLE (31), and other authors have also shown that this reduction was positively correlated with increased egress and retention of these cells in inflamed tissues, mainly the kidney (80). The low number of these monocytes may affect their patrolling function, indirectly contributing to a reduction in endothelial patrol and to the increased number of circulating MPs observed in patients with SLE. In addition, high doses of glucocorticoids (500 mg/day of methylprednisolone) decrease the number of CD14 + CD16 + monocytes in HCs (81). In the present study, we classified patients according to glucocorticoid doses as well as doses of another type of immunosuppressive medication; however, we did not observe differences in the frequency and number of monocyte subsets using these criteria (DNS).

Based on our evidence, MPs and monocyte subsets from patients with SLE display differential expression of surface markers and receptors in a manner dependent on disease activity. Our current results indicate an imbalance in putative MP receptors that promote inflammatory responses (CD64 and CD18) versus non-inflammatory responses (CD36 and CD35) on monocyte subsets in patients with aSLE. In addition, patients display increased levels of PMPs, an important source of ICs that can directly activate monocytes. Therefore, we propose that these vesicles participate in SLE pathogenesis by activating these cells, and these changes in the MPs and monocyte phenotype should be evaluated in future studies as potential biomarkers of disease activity or the treatment response.

## Ethics Statement

Our study complies with the Declaration of Helsinki; the research protocol and informed consent form were approved by the Universidad de Antioquia’s Medical Research Institute and HUSVF Ethics Committees. All patients and HCs provided consent and signed the informed consent form.

## Author Contributions

CB, MR, and DC contributed to the study design; acquisition, analysis, and interpretation of the data; and drafting of the paper. JV-V and JO carried out the data acquisition and critically revised the manuscript. CM, AV, and GV carried out the acquisition and interpretation of the clinical data, and critically revised the manuscript. All authors approved the final version of the manuscript and agreed to be accountable for all aspects of the work.

## Conflict of Interest Statement

The authors declare the absence of any commercial or financial relationships that could be construed as a potential conflict of interest.

## References

[1] Jancar S Sanchez Crespo M. Immune complex-mediated tissue injury: a multistep paradigm. Trends Immunol (2005) 26(1):48–55. 10.1016/j.it.2004.11.007 15629409

[2] Bonora M Wieckowsk MR Chinopoulos C Kepp O Kroemer G Galluzzi L Molecular mechanisms of cell death: central implication of ATP synthase in mitochondrial permeability transition. Oncogene (2015) 34(12):1608. 10.1038/onc.2014.462 25790189

[3] van Bavel CC Dieker J Muller S Briand JP Monestier M Berden JH Apoptosis-associated acetylation on histone H2B is an epitope for lupus autoantibodies. Mol Immunol (2009) 47(2–3):511–6. 10.1016/j.molimm.2009.08.009 19747733

[4] Nielsen CT Ostergaard O Stener L Iversen LV Truedsson L Gullstrand B Increased IgG on cell-derived plasma microparticles in systemic lupus erythematosus is associated with autoantibodies and complement activation. Arthritis Rheum (2012) 64(4):1227–36. 10.1002/art.34381 22238051

[5] Sisirak V Sally B D’Agati V Martinez-Ortiz W Ozcakar ZB David J Digestion of chromatin in apoptotic cell microparticles prevents autoimmunity. Cell (2016) 166(1):88–101. 10.1016/j.cell.2016.05.034 27293190 PMC5030815

[6] Morel O Morel N Jesel L Freyssinet JM Toti F. Microparticles: a critical component in the nexus between inflammation, immunity, and thrombosis. Semin Immunopathol (2011) 33(5):469–86. 10.1007/s00281-010-0239-3 21866419

[7] Vasina EM Cauwenberghs S Feijge MA Heemskerk JW Weber C Koenen RR. Microparticles from apoptotic platelets promote resident macrophage differentiation. Cell Death Dis (2011) 2:e211. 10.1038/cddis.2011.94 21956548 PMC3186911

[8] Poon IK Lucas CD Rossi AG Ravichandran KS. Apoptotic cell clearance: basic biology and therapeutic potential. Nat Rev Immunol (2014) 14(3):166–80. 10.1038/nri3607 24481336 PMC4040260

[9] Mohning MP Thomas SM Barthel L Mould KJ McCubbrey AL Frasch SC Phagocytosis of microparticles by alveolar macrophages during acute lung injury requires MerTK. Am J Physiol Lung Cell Mol Physiol (2017) 314:L69–82. 10.1152/ajplung.00058.2017 28935638 PMC6335009

[10] Bardelli C Amoruso A Federici Canova D Fresu L Balbo P Neri T Autocrine activation of human monocyte/macrophages by monocyte-derived microparticles and modulation by PPARgamma ligands. Br J Pharmacol (2012) 165(3):716–28. 10.1111/j.1476-5381.2011.01593.x 21745193 PMC3315043

[11] Niessen A Heyder P Krienke S Blank N Tykocinski LO Lorenz HM Apoptotic-cell-derived membrane microparticles and IFN-alpha induce an inflammatory immune response. J Cell Sci (2015) 128(14):2443–53. 10.1242/jcs.162735 26034070

[12] Angelot F Seilles E Biichle S Berda Y Gaugler B Plumas J Endothelial cell-derived microparticles induce plasmacytoid dendritic cell maturation: potential implications in inflammatory diseases. Haematologica (2009) 94(11):1502–12. 10.3324/haematol.2009.010934 19648164 PMC2770960

[13] Nielsen CT. Circulating microparticles in systemic lupus erythematosus. Dan Med J (2012) 59(11):B4548. 23171755

[14] Antwi-Baffour S Kholia S Aryee YK Ansa-Addo EA Stratton D Lange S Human plasma membrane-derived vesicles inhibit the phagocytosis of apoptotic cells – possible role in SLE. Biochem Biophys Res Commun (2010) 398(2):278–83. 10.1016/j.bbrc.2010.06.079 20599722

[15] Khan F Ali R. Antibodies against nitric oxide damaged poly L-tyrosine and 3-nitrotyrosine levels in systemic lupus erythematosus. J Biochem Mol Biol (2006) 39(2):189–96. 16584635 10.5483/bmbrep.2006.39.2.189

[16] Rother N Pieterse E Lubbers J Hilbrands L van der Vlag J. Acetylated histones in apoptotic microparticles drive the formation of neutrophil extracellular traps in active lupus nephritis. Front Immunol (2017) 8:1136. 10.3389/fimmu.2017.01136 28959262 PMC5604071

[17] Ullal AJ Reich CF III Clowse M Criscione-Schreiber LG Tochacek M Monestier M Microparticles as antigenic targets of antibodies to DNA and nucleosomes in systemic lupus erythematosus. J Autoimmun (2011) 36(3–4):173–80. 10.1016/j.jaut.2011.02.001 21376534

[18] Avalos I Rho YH Chung CP Stein CM. Atherosclerosis in rheumatoid arthritis and systemic lupus erythematosus. Clin Exp Rheumatol (2008) 26(5 Suppl 51):S5–13. 19026140

[19] Soop A Hallstrom L Frostell C Wallen H Mobarrez F Pisetsky DS. Effect of lipopolysaccharide administration on the number, phenotype and content of nuclear molecules in blood microparticles of normal human subjects. Scand J Immunol (2013) 78(2):205–13. 10.1111/sji.12076 23679665

[20] Joerger-Messerli MS Hoesli IM Rusterholz C Lapaire O. Stimulation of monocytes by placental microparticles involves toll-like receptors and nuclear factor kappa-light-chain-enhancer of activated B cells. Front Immunol (2014) 5:173. 10.3389/fimmu.2014.00173 24782870 PMC3995043

[21] Dieker J Tel J Pieterse E Thielen A Rother N Bakker M Circulating apoptotic microparticles in systemic lupus erythematosus patients drive the activation of dendritic cell subsets and prime neutrophils for NETosis. Arthritis Rheumatol (2016) 68(2):462–72. 10.1002/art.39417 26360137

[22] Wener MH Mannik M Schwartz MM Lewis EJ. Relationship between renal pathology and the size of circulating immune complexes in patients with systemic lupus erythematosus. Medicine (Baltimore) (1987) 66(2):85–97. 10.1097/00005792-198703000-00001 3102894

[23] Sasaki T Muryoi T Hatakeyama A Suzuki M Sato H Seino J Circulating anti-DNA immune complexes in active lupus nephritis. Am J Med (1991) 91(4):355–62. 10.1016/0002-9343(91)90152-N 1951379

[24] Bave U Magnusson M Eloranta ML Perers A Alm GV Ronnblom L. Fc gamma RIIa is expressed on natural IFN-alpha-producing cells (plasmacytoid dendritic cells) and is required for the IFN-alpha production induced by apoptotic cells combined with lupus IgG. J Immunol (2003) 171(6):3296–302. 10.4049/jimmunol.171.6.3296 12960360

[25] van Bavel CC Dieker JW Kroeze Y Tamboer WP Voll R Muller S Apoptosis-induced histone H3 methylation is targeted by autoantibodies in systemic lupus erythematosus. Ann Rheum Dis (2011) 70(1):201–7. 10.1136/ard.2010.129320 20699234

[26] Takai T Roles of Fc receptors in autoimmunity. Nat Rev Immunol (2002) 2(8):580–92. 10.1038/nri856 12154377

[27] Ehrenstein MR Notley CA. The importance of natural IgM: scavenger, protector and regulator. Nat Rev Immunol (2010) 10(11):778–86. 10.1038/nri2849 20948548

[28] Bohlson SS O’Conner SD Hulsebus HJ Ho MM Fraser DA. Complement, c1q, and c1q-related molecules regulate macrophage polarization. Front Immunol (2014) 5:402. 10.3389/fimmu.2014.00402 25191325 PMC4139736

[29] Ziegler-Heitbrock L Ancuta P Crowe S Dalod M Grau V Hart DN Nomenclature of monocytes and dendritic cells in blood. Blood (2010) 116(16):e74–80. 10.1182/blood-2010-02-258558 20628149

[30] Yassin LM Rojas M Ramirez LA Garcia LF Vasquez G. Monocyte activation by apoptotic cells removal in systemic lupus erythematosus patients. Cell Immunol (2010) 266(1):52–60. 10.1016/j.cellimm.2010.08.012 20863485

[31] Burbano C Vasquez G Rojas M A modulatory effect of CD14+CD16++ monocytes on CD14++Cd16- monocytes: a possible explanation of monocyte alterations in systemic lupus erythematosus. Arthritis Rheumatol (2014) 66(12):3371–81. 10.1002/art.38860 25168844

[32] Hochberg MC Updating the American College of Rheumatology revised criteria for the classification of systemic lupus erythematosus. Arthritis Rheum (1997) 40(9):1725 10.1002/art.1780400928 9324032

[33] Griffiths B Mosca M Gordon C. Assessment of patients with systemic lupus erythematosus and the use of lupus disease activity indices. Best Pract Res Clin Rheumatol (2005) 19(5):685–708. 10.1016/j.berh.2005.03.010 16150398

[34] Aletaha D Neogi T Silman AJ Funovits J Felson DT Bingham CO III 2010 rheumatoid arthritis classification criteria: an American college of Rheumatology/European league against rheumatism collaborative initiative. Ann Rheum Dis (2010) 69(9):1580–8. 10.1136/ard.2010.138461 20699241

[35] Zhao X Okeke NL Sharpe O Batliwalla FM Lee AT Ho PP Circulating immune complexes contain citrullinated fibrinogen in rheumatoid arthritis. Arthritis Res Ther (2008) 10(4):R94. 10.1186/ar2478 18710572 PMC2575608

[36] Abeles RD McPhail MJ Sowter D Antoniades CG Vergis N Vijay GK CD14, CD16 and HLA-DR reliably identifies human monocytes and their subsets in the context of pathologically reduced HLA-DR expression by CD14(hi)/CD16(neg) monocytes: expansion of CD14(hi)/CD16(pos) and contraction of CD14(lo)/CD16(pos) monocytes in acute liver failure. Cytometry A (2012) 81(10):823–34. 10.1002/cyto.a.22104 22837127

[37] Ziegler A Simon S Lee G. Comminution of carbohydrate and protein microparticles on firing in a ballistic powder injector. J Pharm Sci (2010) 99(12):4917–27. 10.1002/jps.22213 20575004

[38] Mobarrez F Vikerfors A Gustafsson JT Gunnarsson I Zickert A Larsson A Microparticles in the blood of patients with systemic lupus erythematosus (SLE): phenotypic characterization and clinical associations. Sci Rep (2016) 6:36025. 10.1038/srep36025 27777414 PMC5078765

[39] Nagahama M Nomura S Kanazawa S Ozaki Y Kagawa H Fukuhara S. Significance of anti-oxidized LDL antibody and monocyte-derived microparticles in anti-phospholipid antibody syndrome. Autoimmunity (2003) 36(3):125–31. 10.1080/0891693031000079257 12911278

[40] McCarthy EM Moreno-Martinez D Wilkinson FL McHugh NJ Bruce IN Pauling JD Microparticle subpopulations are potential markers of disease progression and vascular dysfunction across a spectrum of connective tissue disease. BBA Clin (2017) 7:16–22. 10.1016/j.bbacli.2016.11.003 28053878 PMC5199156

[41] Nielsen CT Ostergaard O Johnsen C Jacobsen S Heegaard NH. Distinct features of circulating microparticles and their relationship to clinical manifestations in systemic lupus erythematosus. Arthritis Rheum (2011) 63(10):3067–77. 10.1002/art.30499 21702008

[42] Jayachandran M Miller VM Heit JA Owen WG. Methodology for isolation, identification and characterization of microvesicles in peripheral blood. J Immunol Methods (2012) 375(1–2):207–14. 10.1016/j.jim.2011.10.012 22075275 PMC3253871

[43] Stockinger H Gadd SJ Eher R Majdic O Schreiber W Kasinrerk W Molecular characterization and functional analysis of the leukocyte surface protein CD31. J Immunol (1990) 145(11):3889–97. 1700999

[44] Metzelaar MJ Korteweg J Sixma JJ Nieuwenhuis HK. Biochemical characterization of PECAM-1 (CD31 antigen) on human platelets. Thromb Haemost (1991) 66(6):700–7. 1796415

[45] Pierelli L Bonanno G Rutella S Marone M Scambia G Leone G. CD105 (endoglin) expression on hematopoietic stem/progenitor cells. Leuk Lymphoma (2001) 42(6):1195–206. 10.3109/10428190109097744 11911400

[46] Dagur PK Tatlici G Gourley M Samsel L Raghavachari N Liu P CD146+ T lymphocytes are increased in both the peripheral circulation and in the synovial effusions of patients with various musculoskeletal diseases and display pro-inflammatory gene profiles. Cytometry B Clin Cytom (2010) 78(2):88–95. 10.1002/cyto.b.20502 19834966 PMC2904479

[47] Liu L Shi GP CD31: beyond a marker for endothelial cells. Cardiovasc Res (2012) 94(1):3–5. 10.1093/cvr/cvs108 22379038

[48] Michel M Lee K Piette JC Fromont P Schaeffer A Bierling P Platelet autoantibodies and lupus-associated thrombocytopenia. Br J Haematol (2002) 119(2):354–8. 10.1046/j.1365-2141.2002.03817.x 12406068

[49] Hasselaar P Derksen RH Blokzijl L Hessing M Nieuwenhuis HK Bouma BN Risk factors for thrombosis in lupus patients. Ann Rheum Dis (1989) 48(11):933–40. 10.1136/ard.48.11.933 2512863 PMC1003917

[50] Pretorius E du Plooy J Soma P Gasparyan AY. An ultrastructural analysis of platelets, erythrocytes, white blood cells, and fibrin network in systemic lupus erythematosus. Rheumatol Int (2014) 34(7):1005–9. 10.1007/s00296-013-2817-x 23832292

[51] Boilard E Blanco P Nigrovic PA. Platelets: active players in the pathogenesis of arthritis and SLE. Nat Rev Rheumatol (2012) 8(9):534–42. 10.1038/nrrheum.2012.118 22868927

[52] Boudreau LH Duchez AC Cloutier N Soulet D Martin N Bollinger J Platelets release mitochondria serving as substrate for bactericidal group IIA-secreted phospholipase A2 to promote inflammation. Blood (2014) 124(14):2173–83. 10.1182/blood-2014-05-573543 25082876 PMC4260364

[53] Holdenrieder S Stieber P. Clinical use of circulating nucleosomes. Crit Rev Clin Lab Sci (2009) 46(1):1–24. 10.1080/10408360802485875 19107649

[54] Fuchs TA Bhandari AA Wagner DD. Histones induce rapid and profound thrombocytopenia in mice. Blood (2011) 118(13):3708–14. 10.1182/blood-2011-01-332676 21700775 PMC3186342

[55] Semeraro F Ammollo CT Morrissey JH Dale GL Friese P Esmon NL Extracellular histones promote thrombin generation through platelet-dependent mechanisms: involvement of platelet TLR2 and TLR4. Blood (2011) 118(7):1952–61. 10.1182/blood-2011-03-343061 21673343 PMC3158722

[56] Sellam J Proulle V Jungel A Ittah M Miceli Richard C Gottenberg JE Increased levels of circulating microparticles in primary Sjogren’s syndrome, systemic lupus erythematosus and rheumatoid arthritis and relation with disease activity. Arthritis Res Ther (2009) 11(5):R156 10.1186/ar2833 19832990 PMC2787287

[57] Dhir V Singh AP Aggarwal A Naik S Misra R. Increased T-lymphocyte apoptosis in lupus correlates with disease activity and may be responsible for reduced T-cell frequency: a cross-sectional and longitudinal study. Lupus (2009) 18(9):785–91. 10.1177/0961203309103152 19578102

[58] Hatterer E Shang L Simonet P Herren S Daubeuf B Teixeira S A specific anti-citrullinated protein antibody profile identifies a group of rheumatoid arthritis patients with a toll-like receptor 4-mediated disease. Arthritis Res Ther (2016) 18(1):224. 10.1186/s13075-016-1128-5 27716430 PMC5053084

[59] Rekvig OP The anti-DNA antibody: origin and impact, dogmas and controversies. Nat Rev Rheumatol (2015) 11(9):530–40. 10.1038/nrrheum.2015.69 26034836

[60] Rekvig OP Anti-dsDNA antibodies as a classification criterion and a diagnostic marker for systemic lupus erythematosus: critical remarks. Clin Exp Immunol (2015) 179(1):5–10. 10.1111/cei.12296 24533624 PMC4260890

[61] Ferracini M Rios FJ Pecenin M Jancar S. Clearance of apoptotic cells by macrophages induces regulatory phenotype and involves stimulation of CD36 and platelet-activating factor receptor. Mediators Inflamm (2013) 2013:950273. 10.1155/2013/950273 24347838 PMC3854564

[62] Kreimer S Belov AM Ghiran I Murthy SK Frank DA Ivanov AR. Mass-spectrometry-based molecular characterization of extracellular vesicles: lipidomics and proteomics. J Proteome Res (2015) 14(6):2367–84. 10.1021/pr501279t 25927954

[63] Li Y Lee PY Kellner ES Paulus M Switanek J Xu Y Monocyte surface expression of Fcgamma receptor RI (CD64), a biomarker reflecting type-I interferon levels in systemic lupus erythematosus. Arthritis Res Ther (2010) 12(3):R90. 10.1186/ar3017 20478071 PMC2911874

[64] Kikuchi-Taura A Yura A Tsuji S Ohshima S Kitatoube A Shimizu T Monocyte CD64 expression as a novel biomarker for the disease activity of systemic lupus erythematosus. Lupus (2015) 24(10):1076–80. 10.1177/0961203315579093 25804673

[65] Banchereau J Pascual V. Type I interferon in systemic lupus erythematosus and other autoimmune diseases. Immunity (2006) 25(3):383–92. 10.1016/j.immuni.2006.08.010 16979570

[66] Kyogoku C Smiljanovic B Grun JR Biesen R Schulte-Wrede U Haupl T Cell-specific type I IFN signatures in autoimmunity and viral infection: what makes the difference? PLoS One (2013) 8(12):e83776. 10.1371/journal.pone.0083776 24391825 PMC3877094

[67] Lovgren T Eloranta ML Kastner B Wahren-Herlenius M Alm GV Ronnblom L Induction of interferon-alpha by immune complexes or liposomes containing systemic lupus erythematosus autoantigen- and Sjogren’s syndrome autoantigen-associated RNA. Arthritis Rheum (2006) 54(6):1917–27. 10.1002/art.21893 16729300

[68] Clarke EV Weist BM Walsh CM Tenner AJ. Complement protein C1q bound to apoptotic cells suppresses human macrophage and dendritic cell-mediated Th17 and Th1 T cell subset proliferation. J Leukoc Biol (2015) 97(1):147–60. 10.1189/jlb.3A0614-278R 25381385 PMC4377823

[69] Chen Y Park YB Patel E Silverman GJ IgM antibodies to apoptosis-associated determinants recruit C1q and enhance dendritic cell phagocytosis of apoptotic cells. J Immunol (2009) 182(10):6031–43. 10.4049/jimmunol.0804191 19414754 PMC4428684

[70] Albert ML Death-defying immunity: do apoptotic cells influence antigen processing and presentation? Nat Rev Immunol (2004) 4(3):223–31. 10.1038/nri11308 15039759

[71] Ostergaard O Nielsen CT Iversen LV Tanassi JT Knudsen S Jacobsen S Unique protein signature of circulating microparticles in systemic lupus erythematosus. Arthritis Rheum (2013) 65(10):2680–90. 10.1002/art.38065 23817959

[72] Wolf D Bukosza N Engel D Poggi M Jehle F Anto Michel N Inflammation, but not recruitment, of adipose tissue macrophages requires signalling through Mac-1 (CD11b/CD18) in diet-induced obesity (DIO). Thromb Haemost (2017) 117(2):325–38. 10.1160/TH16-07-0553 27853810

[73] Adib M Ragimbeau J Avrameas S Ternynck T. IgG autoantibody activity in normal mouse serum is controlled by IgM. J Immunol (1990) 145(11):3807–13. 2246515

[74] Avrameas S. Natural autoantibodies: from ‘horror autotoxicus’ to ‘gnothi seauton’. Immunol Today (1991) 12(5):154–9. 10.1016/S0167-5699(05)80045-3 1715166

[75] Forger F Matthias T Oppermann M Becker H Helmke K. Clinical significance of anti-dsDNA antibody isotypes: IgG/IgM ratio of anti-dsDNA antibodies as a prognostic marker for lupus nephritis. Lupus (2004) 13(1):36–44. 10.1191/0961203304lu485oa 14870916

[76] Litvack ML Post M Palaniyar N. IgM promotes the clearance of small particles and apoptotic microparticles by macrophages. PLoS One (2011) 6(3):e17223. 10.1371/journal.pone.0017223 21448268 PMC3063157

[77] Chen Y Khanna S Goodyear CS Park YB Raz E Thiel S Regulation of dendritic cells and macrophages by an anti-apoptotic cell natural antibody that suppresses TLR responses and inhibits inflammatory arthritis. J Immunol (2009) 183(2):1346–59. 10.4049/jimmunol.0900948 19564341 PMC2713016

[78] Lang KS Lang PA Meryk A Pandyra AA Boucher LM Pozdeev VI Involvement of Toso in activation of monocytes, macrophages, and granulocytes. Proc Natl Acad Sci U S A (2013) 110(7):2593–8. 10.1073/pnas.1222264110 23359703 PMC3574925

[79] Cros J Cagnard N Woollard K Patey N Zhang SY Senechal B Human CD14dim monocytes patrol and sense nucleic acids and viruses via TLR7 and TLR8 receptors. Immunity (2010) 33(3):375–86. 10.1016/j.immuni.2010.08.012 20832340 PMC3063338

[80] Barrera Garcia A Gomez-Puerta JA Arias LF Burbano C Restrepo M Vanegas AL Infiltrating CD16+ are associated with a reduction in peripheral CD14+CD16++ monocytes and severe forms of lupus nephritis. Autoimmune Dis (2016) 2016:9324315. 10.1155/2016/9324315 28070418 PMC5187455

[81] Fingerle-Rowson G Angstwurm M Andreesen R Ziegler-Heitbrock HW. Selective depletion of CD14+ CD16+ monocytes by glucocorticoid therapy. Clin Exp Immunol (1998) 112(3):501–6. 10.1046/j.1365-2249.1998.00617.x 9649222 PMC1904988

