# Supplementary material for: Potential Involvement of Platelet-Derived Microparticles and Microparticles Forming Immune Complexes during Monocyte Activation in Patients with Systemic Lupus Erythematosus
Source: Front Immunol. 2018 Mar 1;9:322. doi: 10.3389/fimmu.2018.00322 (PMC5837989; doi:10.3389/fimmu.2018.00322)
Supplement: Supplementary file 1 [file image_1.pdf]

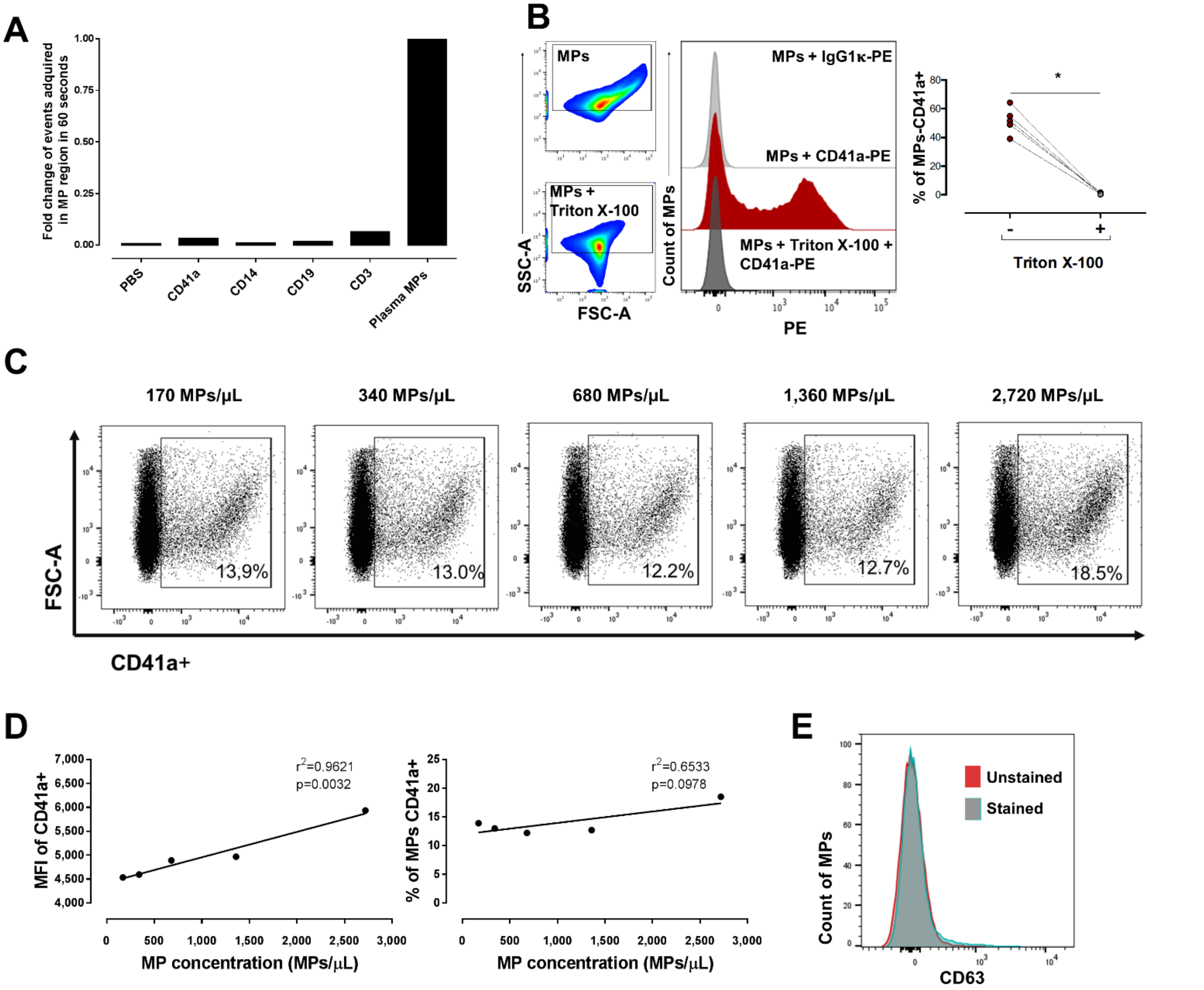

Supplementary Figure 1. Acquisition controls for the MP analysis. (A) Controls for buffer and antibody aggregation. Representative number of events observed in DPBS and the antibodies (against to CD3, CD14, CD19, and CD41a) alone acquired in the flow cytometer for 1 min using the MP parameters. Results from at least two independent experiments are shown. Only minimal noise or non-specific signals for the buffer and antibodies were observed compared with the number of MPs detected in unlabeled plasma samples (diluted in DPBS). Fold changes were calculated dividing the number of events detected in each sample by the number of events detected in MPs isolated from plasma. **(B)** MPs are sensitive to detergent treatment. Representative SSC-A versus FSC-A density diagrams of untreated (upper left panel), and 0.05% Triton X-100-treated MPs (lower left panel) (György B et al. (2011) Blood, 117:e39-48). Representative histograms show CD41a expression on MPs treated with and without the detergent (middle panel). Percentages of untreated and Triton X-100-treated MPs-CD41a+ (right panel). Mann-Whitney U test, **p ≤0.01, n=5. The SSC-A and FSC-A parameters and CD41a positivity were rapidly altered by the Triton X-100 treatment; a finding consistent with the lipid membrane composition described for MPs. **(C)**

Representative dot plots of plasma MPs from a healthy individual stained with anti-human CD41a and acquired at different concentrations by flow cytometry using equal parameters and low flow conditions. The results from at least two independent experiments are shown. **(D)** Regression curves show the frequency of MPs-CD41a+ (right panel) and the mean fluorescence intensity (MFI, left panel) of this molecule versus the MP concentrations. These data were obtain as described in C. The results from at least two independent experiments are shown. Optimization of the MP content for the staining procedures was performed in a preliminary phase of this research, in which we determined that 200 µL of PPP contained the optimal number of MPs to be stained and analysed by flow cytometry. The MPs obtained in this volume were acquired in 300 µL of DPBS, corresponding to a concentration of approximately 350 MPs/µL for HCs, 650 MPs/µL for patients with iSLE and 1000 MPs for patients with aSLE. In addition, we always acquired these vesicles under low flow (low pressure) conditions. **(E)** Representative histogram showing CD63 expression on MPs. This molecule was not detected on circulating MPs in three independent experiments, suggesting that these vesicles were not contaminated with exosomes.
