# Supplementary material for: Potential Involvement of Platelet-Derived Microparticles and Microparticles Forming Immune Complexes during Monocyte Activation in Patients with Systemic Lupus Erythematosus
Source: Front Immunol. 2018 Mar 1;9:322. doi: 10.3389/fimmu.2018.00322 (PMC5837989; doi:10.3389/fimmu.2018.00322)
Supplement: Supplementary file 2 [file image_2.pdf]

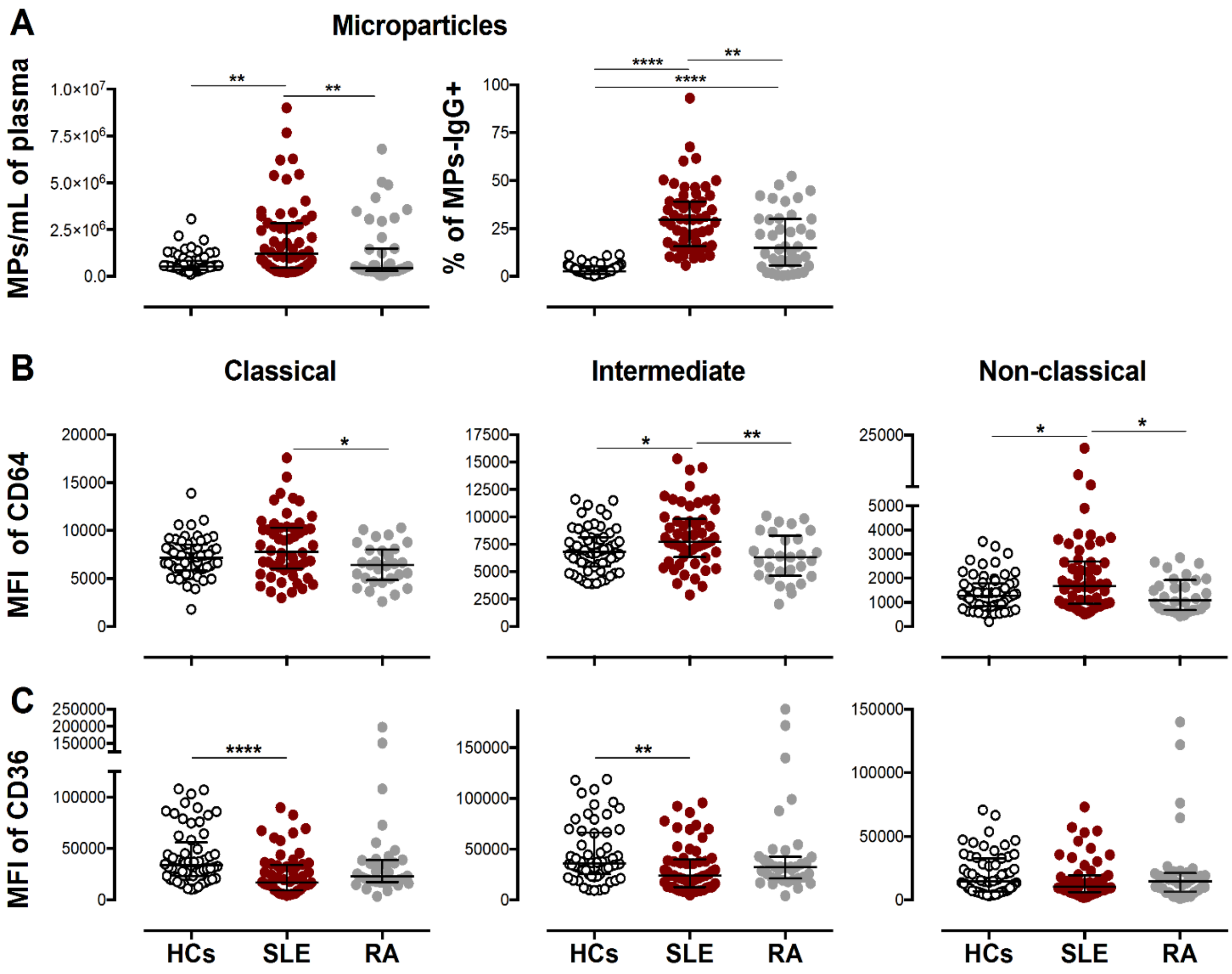

Supplementary Figure 2. Levels of circulating MPs and monocyte subsets in total patients with SLE differ from those in patients with RA. (A) Total MP number (left panel) and frequency (right panel) of circulating MPs-IgG+ in plasma from total patients with SLE (n=60), patients with RA (n=40), and HCs (n=60). The MFI of **(B)** CD64, and **(C)** CD36 on classical, intermediate and non-classical monocytes from patients with SLE (n=60), patients with RA (n=40), and HCs (n=40) are shown. Kruskal-Wallis test and Dunn's *post-hoc* test *$p \leq 0.05$, **$p \leq 0.01$, ***$p \leq 0.001$ and ****$p \leq 0.0001$.
