# Supplementary material for: Potential Involvement of Platelet-Derived Microparticles and Microparticles Forming Immune Complexes during Monocyte Activation in Patients with Systemic Lupus Erythematosus
Source: Front Immunol. 2018 Mar 1;9:322. doi: 10.3389/fimmu.2018.00322 (PMC5837989; doi:10.3389/fimmu.2018.00322)
Supplement: Supplementary file 3 [file image_3.pdf]

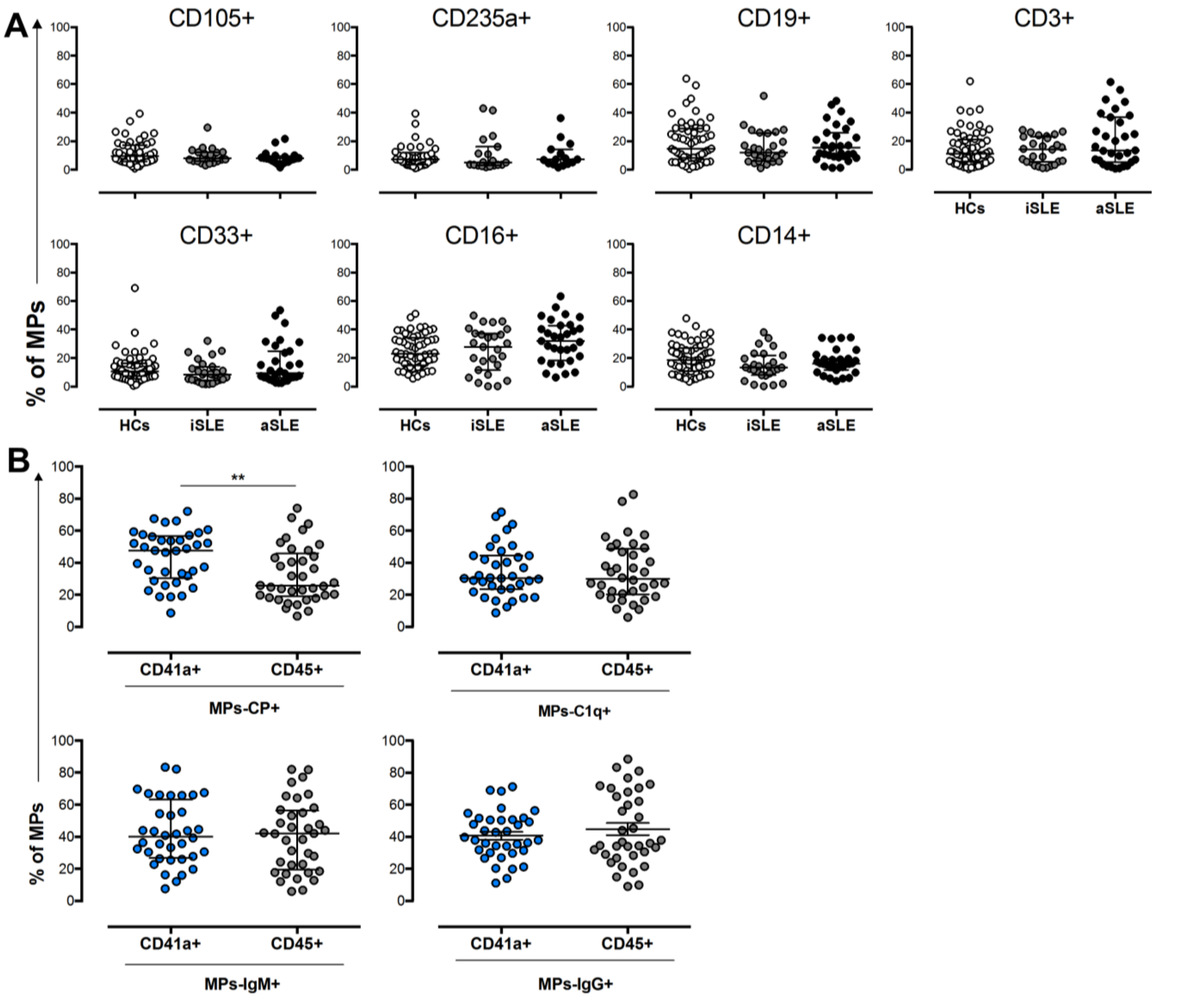

Supplementary Figure 3. Other cell sources of circulating MPs do not differ between patients with SLE and HCs, and patients with SLE display an enrichment of CP on MPs-CD41a+. (A) Frequency of circulating MPs-CD105+, MPs-CD235a+, MPs-CD19+, MPs-CD3+, MPs-CD33+, MPs-CD16+, and MPs-CD14+ in HCs, patients with iSLE and patients with aSLE. Comparisons among the groups were performed using the Kruskal-Wallis test and Dunn's *post-hoc* test. **(B)** Frequency of MPs-CD41a+ and MPs-CD45+ among MPs-CP+ (upper left panel). Frequency of MPs-CD41a+ and MPs-CD45+ among MPs-C1q+ (upper right panel). Frequency of MPs-CD41a+ and MPs-CD45+ among MPs-IgM+ (lower left panel). Frequency of MPs-CD41a+ and MPs-CD45+ among MPs-IgG+ (lower right panel). Comparisons were performed using the Mann-Whitney U test; $**p \leq 0.01$; patients with iSLE, n=28; patients with aSLE, n=32.
