# Supplementary material for: Potential Involvement of Platelet-Derived Microparticles and Microparticles Forming Immune Complexes during Monocyte Activation in Patients with Systemic Lupus Erythematosus
Source: Front Immunol. 2018 Mar 1;9:322. doi: 10.3389/fimmu.2018.00322 (PMC5837989; doi:10.3389/fimmu.2018.00322)
Supplement: Supplementary file 4 [file image_4.pdf]

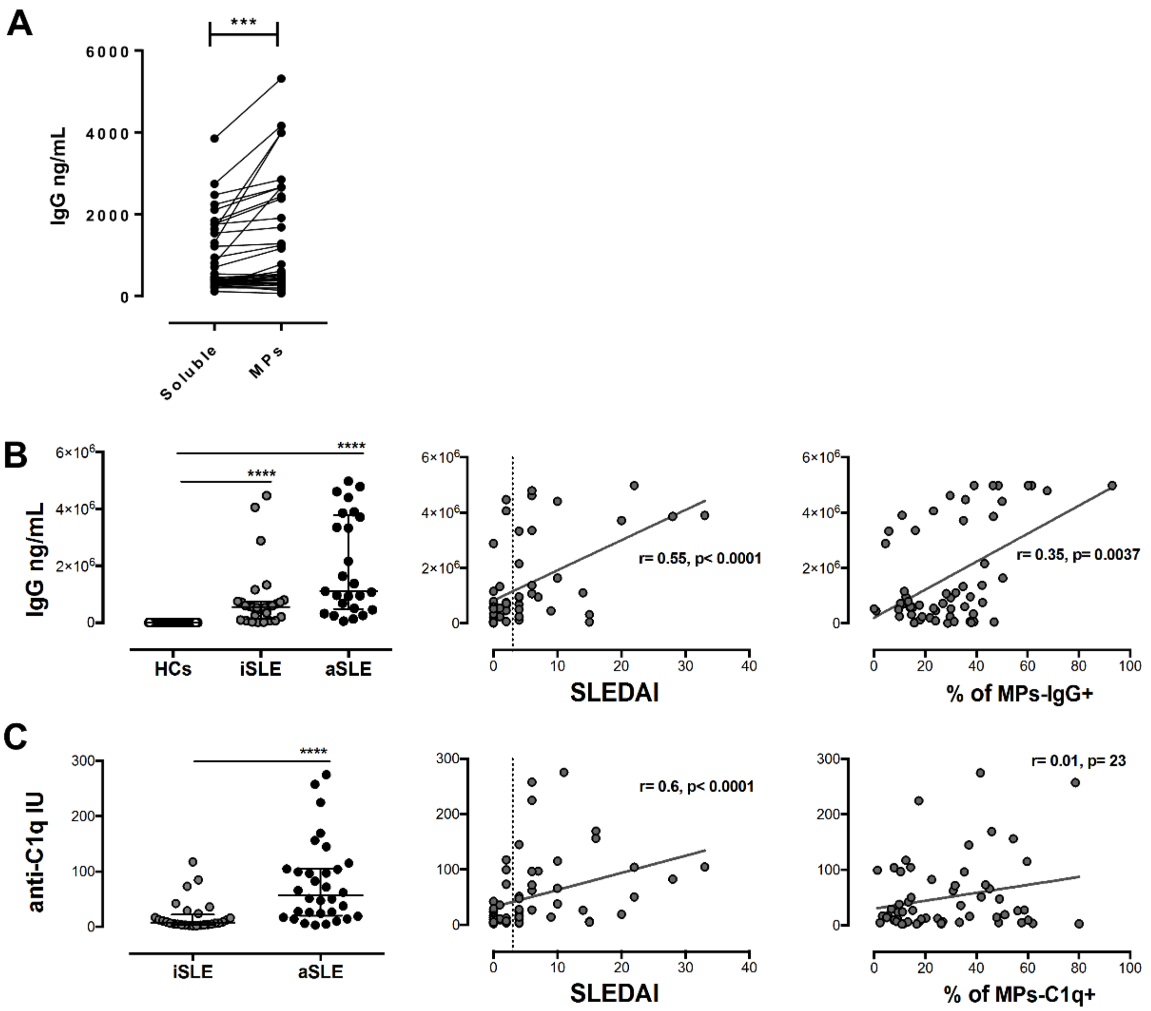

Supplementary Figure 4. Most of the total circulating ICs-IgG+ are located on MPs, and the circulating ICs-IgG+ and serum anti-C1q antibody levels are associated with SLEDAI scores. (A) Comparison of soluble circulating ICs-IgG+ and MPs-IgG+ levels in patients with SLE, as evaluated by ELISA. **(B)** Concentration of total circulating ICs-IgG+ in patients with iSLE, patients with aSLE, and HCs (left panel); analysis of the correlations between total circulating ICs-IgG+ levels with SLEDAI scores (centre panel) and the frequency of MPs-IgG+ evaluated by flow cytometry (right panel). **(C)** Concentration of serum anti-C1q antibodies in patients with iSLE and aSLE (left panel); analysis of the correlations between serum anti-C1q antibody levels with SLEDAI scores (centre panel) and the frequency of MPs-C1q+ (right panel). Comparisons among groups were performed using the Kruskal-Wallis test and Dunn's *post hoc* test ***p ≤0.001, ****p ≤0.0001. Correlation analyses were performed by determining Spearman's rank correlation coefficients and 95% confidence intervals; the dotted line indicates the cut-off point between patients with iSLE (SLEDAI <4, n=28) and those with aSLE (SLEDAI ≥4, n=32).
