# Supplementary material for: Potential Involvement of Platelet-Derived Microparticles and Microparticles Forming Immune Complexes during Monocyte Activation in Patients with Systemic Lupus Erythematosus
Source: Front Immunol. 2018 Mar 1;9:322. doi: 10.3389/fimmu.2018.00322 (PMC5837989; doi:10.3389/fimmu.2018.00322)
Supplement: Supplementary file 5 [file image_5.pdf]

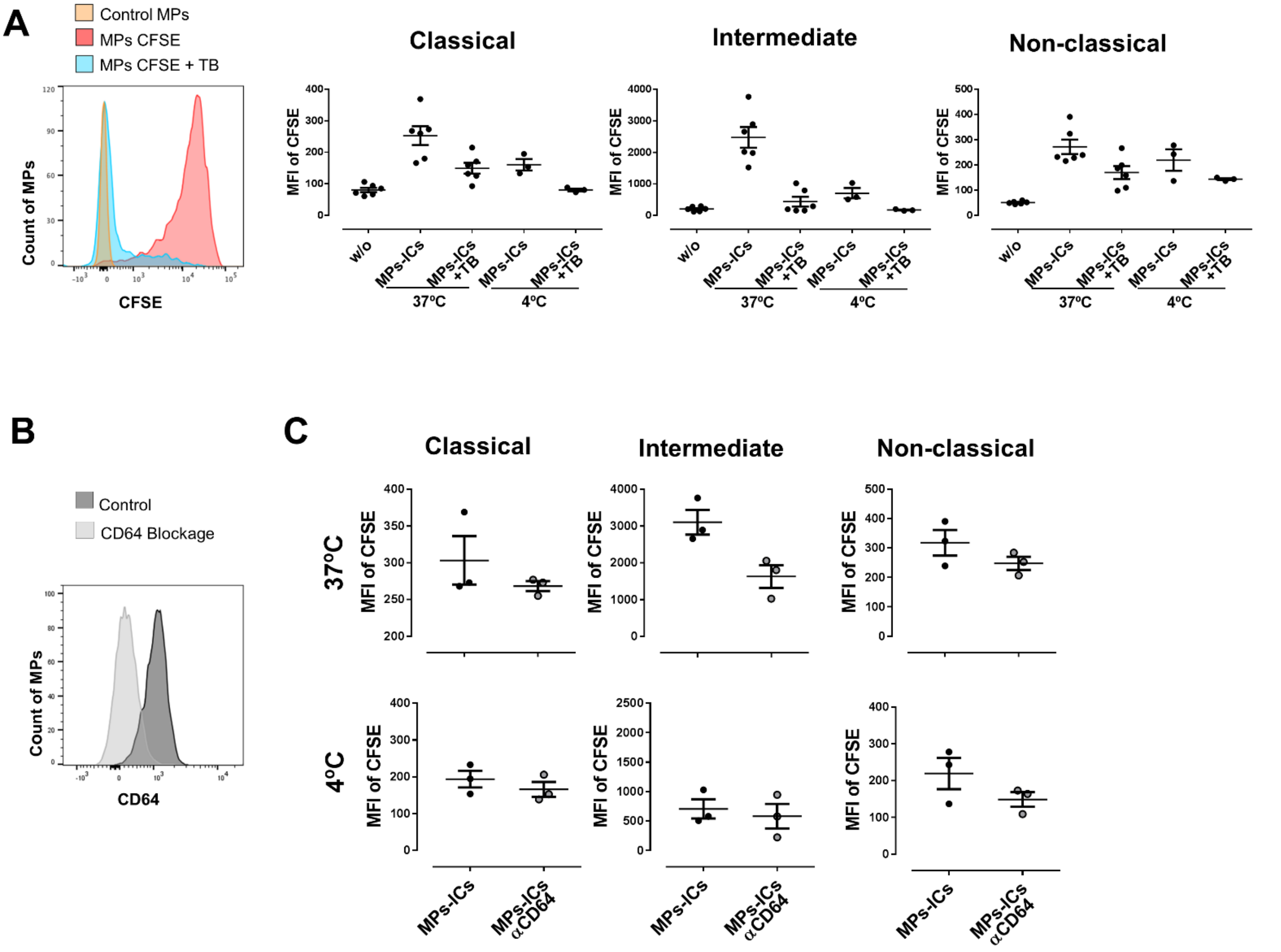

Supplementary Figure 5. Controls for the binding and uptake of MPs and MPs-ICs from patients with SLE. (A) Representative histograms of the CFSE-labelled MPs from a batch of patients with SLE acquired immediately after the staining (5 µM, red histogram), and the same MPs after quenching with trypan blue (TB, blue histogram). The results from at least three independent experiments are shown (left panel). Consolidated results for the binding and uptake of MPs-ICs from patients with SLE by monocyte subsets from HCs at 37°C and 4°C. n=3-6 (right panel). A higher CSFE signal was detected in monocyte subsets when MPs-ICs were incubated at 37°C (bound and internalized MPs) than at 4°C (bound MPs only). Thus, the additional CSFE signal at 37°C corresponded to the events inside monocyte subsets. In addition, the remaining signal observed after MPs-ICs were quenched with TB and incubated at 37°C must correspond to the MPs-ICs inside cells; in contrast, when this experiment was performed at 4°C, the signal was similar to the values observed in control cells, mainly for classical and intermediate monocytes. w/o (without any treatment). Based on these findings, the main mechanism by which these vesicles are internalized is mediated by an endocytic pathway, as previously suggested by other researchers (Morelli. *et al*. (2004) Blood 104, 3257–3266; Mohning, M. P., *et al.* (2017). Am J Physiol Lung Cell Mol Physiol, ajplung 00058 02017). However, this mechanism was not as clear for non-classical monocytes; suggesting

that in these cells, MPs is also internalized by non-specific pathways, such as membranes fusion (Al-Nedawi, K. *et al.* (2008). Nature Cell Biol. 10, 619–624; Skog, J. *et al.* (2008). Nature Cell Biol. 10, 1470–1476; Ratajczak, J. *et al.* (2006). Leukaemia 20, 847–856). In addition, the signal of bound MPs-ICs at 4°C was always lower than the signal of bound MPs-ICs at 37°C after the TB treatment. This finding may be explained by the observation that more membrane fusion would occur at 37°C than at 4°C. However, additional, more detailed experiments are required to investigate this hypothesis. **(B)** CD64 blocking control (αCD64). PBMCs from HCs were blocked with or without a specific antibody, as described in the Materials and Methods section. Thereafter, this receptor was detected by flow cytometry. CD64 blockade affected the detection of this molecule. The results from at least two independent experiments are shown. **(C)** The binding and uptake of MPs-ICs were not affected by CD64 blockade. Consolidated data for the binding and uptake of MPs-ICs from patients with SLE by monocyte subpopulations from HCs treated with or without αCD64 are shown. n=3.
