# Supplementary material for: Potential Involvement of Platelet-Derived Microparticles and Microparticles Forming Immune Complexes during Monocyte Activation in Patients with Systemic Lupus Erythematosus
Source: Front Immunol. 2018 Mar 1;9:322. doi: 10.3389/fimmu.2018.00322 (PMC5837989; doi:10.3389/fimmu.2018.00322)
Supplement: Supplementary file 6 [file image_6.pdf]

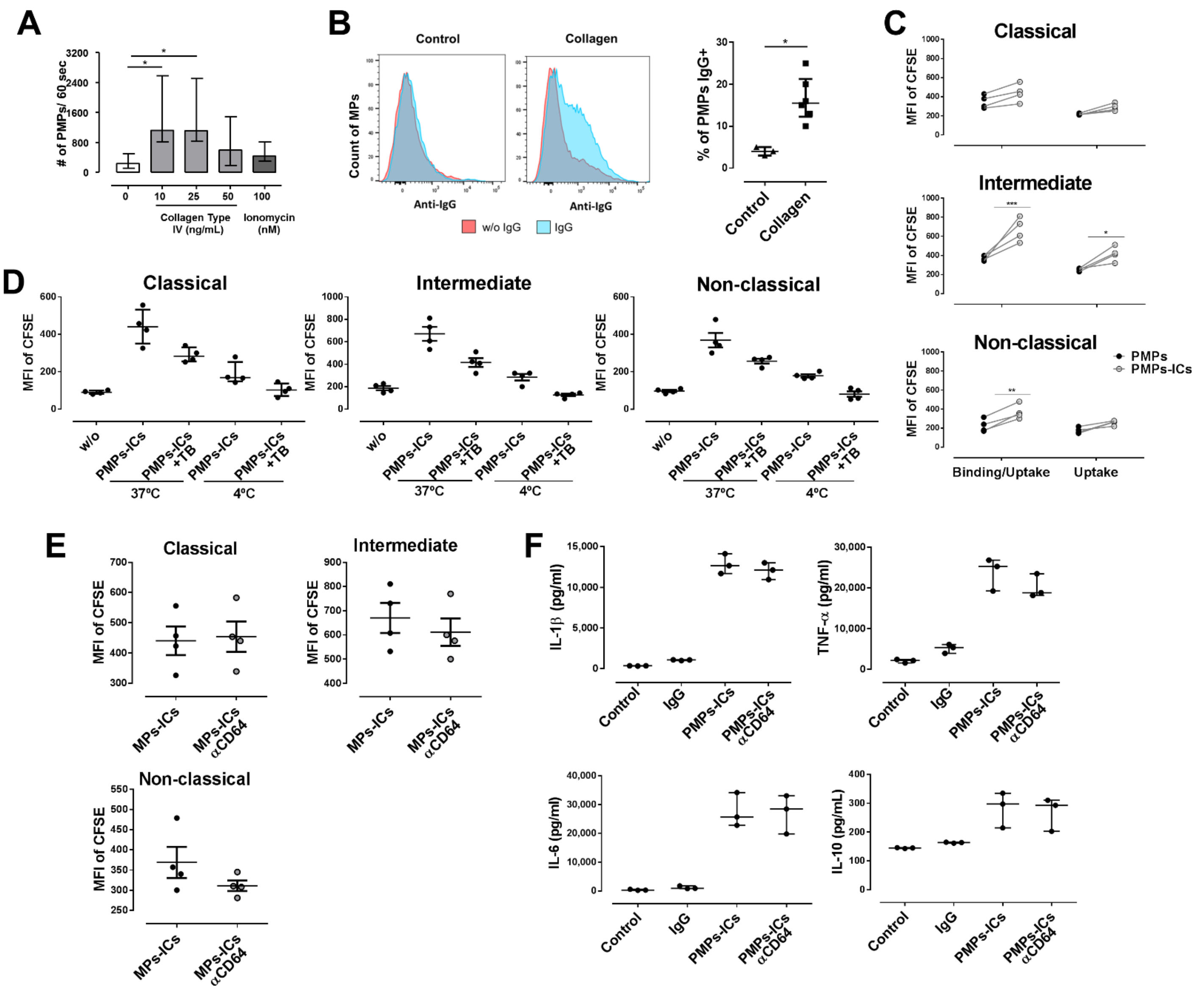

Supplementary Figure 6. The number of PMPs increases after collagen stimulation of platelets; these structures form immune complexes (ICs) with IgG from patients with SLE and are bound and taken up by monocytes from HCs in a CD64-independent manner. (A) Number of PMPs after the culture of platelets from HCs with or without different concentrations of Collagen Type IV. Kruskal-Wallis and Dunn's *post-hoc* tests were performed, n=5. *p≤0.05; results are shown as medians and interquartile ranges. The results correspond to at least 3 independent experiments. **(B)** Representative histograms comparing PMPs generated without stimulation (control) or after treatment with 10 ng/mL Collagen Type IV. These structures were opsonised with IgG from patients with SLE and IC formation was assessed using anti-IgG $F(ab)_2$ staining (left panel). The percentages of PMPs-IgG+ in different PMPs-ICs batches were calculated using the overtone subtraction tool. Consolidated results are shown in the right panel. Mann-Whitney U tests, n=3-6. *p≤0.05; results are shown as medians and interquartile ranges. **(C)** Binding and uptake of PMPs and PMPs-ICs labelled with CFSE by monocyte subsets from HCs. n=4. Two-way ANOVA and Bonferroni's *post-hoc* test. **(D)** Binding and uptake of PMPs-ICs (1:1

vesicle:cell ratio) by monocyte subpopulations from healthy individuals at 37°C and 4°C. n=3-6, w/o (without any treatment). The CSFE signal in monocyte subsets was higher when PMPs-ICs were incubated at 37°C (bound and internalized MPs) than at 4°C (bound MPs only). Thus, the additional CSFE signal observed at 37°C corresponds to the events inside monocyte subsets. In addition, the remaining signal observed after PMPs-ICs were quenched with trypan blue (TB) and incubated at 37°C must correspond to the vesicles inside cells. In contrast, when this experiment was performed at 4°C, the signal was similar to the values observed in control cells after the TB treatment, suggesting that a main mechanism of the internalization of these vesicles is mediated by an endocytic pathway, as previously suggested by other researchers (Morelli. et al. (2004) Blood 104, 3257–3266) (Mohning, M. P., et al. (2017). Am J Physiol Lung Cell Mol Physiol, ajplung 00058 02017). In addition, the signal of bound PMPs-ICs at 4°C was always lower than the signal of bound PMPs-ICs at 37°C after the TB treatment. This finding may be explained by the observation that more membrane fusion would occur at 37°C than at 4°C. However, additional, more detailed experiments are required to investigate this hypothesis. **(E)** Consolidated data for the binding and uptake of PMPs-ICs by monocyte subsets from HCs treated with or without CD64 are shown. n=3. **(F)** IgG from patients with SLE alone does not induce cytokine release, and CD64 blockade does not affect cytokine production induced by PMPs-ICs. Consolidated results for IL1-β, TNF-α, IL-6 and IL-10 concentrations in supernatants from monocytes cultured with IgG from patients with SLE alone, PMPs-ICs and PMPs-ICs with CD64 blockade are shown. n=3. Results from at least two independent experiments are shown.
